# CPL‐Diff: A Diffusion Model for De Novo Design of Functional Peptide Sequences with Fixed Length

**DOI:** 10.1002/advs.202412926

**Published:** 2025-04-15

**Authors:** Zhenjie Luo, Aoyun Geng, Leyi Wei, Quan Zou, Feifei Cui, Zilong Zhang

**Affiliations:** ^1^ College of Computer Science and Technology Hainan University No. 58, Renmin Avenue Haikou 570228 China; ^2^ Centre for Artificial Intelligence driven Drug Discovery Faculty of Applied Science Macao Polytechnic University Macao SAR 999078 China; ^3^ School of Informatics Xiamen University Xiamen 361005 China; ^4^ Institute of Fundamental and Frontier Sciences University of Electronic Science and Technology of China Chengdu 610054 China; ^5^ Yangtze Delta Region Institute (Quzhou) University of Electronic Science and Technology of China Quzhou 324000 China

**Keywords:** artificial intelligent, de novo design, diffusion model, protein language model, therapeutic peptide

## Abstract

Peptides are recognized as next‐generation therapeutic drugs due to their unique properties and are essential for treating human diseases. In recent years, a number of deep generation models for generating peptides have been proposed and have shown great potential. However, these models cannot well control the length of the generated sequence, while the sequence length has a very important impact on the physical and chemical properties and therapeutic effects of peptides. Here, a diffusion model is introduced, capable of controlling the length of generated functional peptide sequences, named CPL‐Diff. CPL‐Diff can control the length of generated polypeptide sequences using only attention masking. Additionally, CPL‐Diff can generate single‐functional polypeptide sequences based on given conditional information. Experiments demonstrate that the peptides generated by CPL‐Diff exhibit lower perplexity and similarity compared to those produced by the current state‐of‐the‐art models, and further exhibit relevant physicochemical properties similar to real sequences. The interpretability analysis is also performed on CPL‐Diff to understand how it controls the length of generated sequences and the decision‐making process involved in generating polypeptide sequences, with the aim of providing important theoretical guidance for polypeptide design. The code for CPL‐Diff is available at https://github.com/luozhenjie1997/CPL‐Diff.

## Graphical abstract

This study presents a diffusion model for generating functional peptide sequence lengths using mask control. The model can generate antimicrobial, antifungal, and antiviral peptides with specific lengths on demand. The model learns the structure of peptides better and generates peptides with better physicochemical properties, and the model has good generalization ability and interpretability.

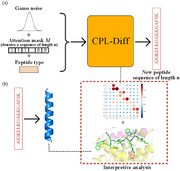

## 1 Introduction

Therapeutic peptides, including antimicrobial peptides (AMPs), antifungal peptides (AFPs), and antiviral peptides (AVPs) are unique medications comprised of short chains of amino acids with great potential in treating complex human diseases.^[^ ^1^ ^]^ These short peptides hold tremendous potential in treating complex human diseases due to their compact structure and strong adaptability, promising to radically alter therapeutic interventions for illnesses caused by bacteria, fungi, parasites, and viruses.^[^ ^2^ ^]^ However, the current engineering paradigms for these peptides are primarily based on high‐throughput screening and rational design aimed at enhancing in vivo stability, solubility, and strain specificity while reducing aggregation.^[^ ^3^ ^]^ Although the flexibility of peptides is advantageous for clinical research, it complicates the design process since traditional structure‐based methods often struggle to handle the dynamic and conformationally unstable nature of these molecules.^[^ ^4^, ^5^ ^]^ Moreover, the combinatorial space of these peptides is huge, of which again only a small fraction of the solutions meets clinical needs. Thus, this approach to screening, which is based on an approximation of an exhaustive method, can be both time‐consuming and costly.

In recent years, deep generative models (DGMs) have achieved good results in generating images^[^ ^6^ ^]^ and text,^[^ ^7^ ^]^ and have gained popularity in protein generation.^[^ ^8^ ^]^ For example, based on autoregressive methods, peptide sequences are depicted as sentences composed of amino acid tokens, so that the problem can be solved by predicting the amino acid arrangement through recursive neural networks (RNNs).^[^ ^9^ ^]^ Methods based on variational autoencoders (VAEs) sample from the latent space learned through the encoder‐decoder architecture to generate new peptide sequences, and treat therapeutic properties as conditional constraints or not as conditional constraints.^[^ ^10^, ^11^ ^]^ Methods based on generative adversarial networks (GANs) use known data to train generators and discriminators, which learn data distribution in a competitive manner, allowing the generator to generate new peptides that are close to the distribution of real peptides.^[^ ^12^ ^]^

The diffusion generative model proposed by Sohl‐Dickstein et al.^[^ ^13^ ^]^ has garnered significant attention due to its remarkable performance in image^[^ ^14^, ^15^ ^]^ and speech generation.^[^ ^16^, ^17^ ^]^ Compared to previous generative model technologies, diffusion models exhibit stronger capabilities in fitting data distributions, with better convergence and more diverse generated samples.^[^ ^10^ ^]^ For example, Vinod et al.^[^ ^18^ ^]^ demonstrated the feasibility of developing a generative diffusion model by optimizing the function of downstream tasks and comparing pure sequence models, pure structure models, and sequence‐structure joint models. TaxDiff^[^ ^19^ ^]^ combines biological species information and the generative ability of diffusion models, inserting classification information into each layer of the Transformer block to achieve fine control, thereby guiding the diffusion model to generate structurally stable proteins in sequence space. MMCD^[^ ^20^ ^]^ integrates both sequence and structure modalities in the diffusion model and aligns the information of these two modalities to enhance the ability of the diffusion model to generate high‐quality therapeutic peptides, jointly generating new peptide sequences and structures.

At the same time, the emergence of protein language models (pLMs) such as ESM‐2,^[^ ^21^ ^]^ ProtT5,^[^ ^22^ ^]^ and ProGen^[^ ^23^ ^]^ has significantly advanced our understanding and design capabilities of proteins. Combining diffusion models with pLMs can further enhance protein design capabilities. For example, ForceGen^[^ ^24^ ^]^ combines ESM2 and uses diffusion models to generate latent space embeddings of protein sequences in ESM2. By using the expected tensile force response curve as a condition, the generated proteins generally better meet the design goals in terms of tensile force response. AMP‐Diffusion^[^ ^25^ ^]^ is the first latent space diffusion pLM, which uses ESM2 8M for its pLM and Tramsformer architecture for its denoising structure to directly predict the original embedding. The generated AMPs show statistical robustness in multiple evaluation metrics and physicochemical properties. ProT‐Diff^[^ ^26^ ^]^ has demonstrated through wet experiments that combining diffusion models and pLM can generate AMPs with strong antibacterial activity, low hemolytic rate cytotoxicity, and broad‐spectrum efficacy.

However, these therapeutic peptide generation models do not consider enforcing control over the length of the generated sequences, and most of the models for generating peptides are trained using only one type of peptide. In fact, the length of the peptide sequence has an important influence on the effect and physical and chemical properties of the peptide. For example, the length of the peptide chain of an AMP affects the hydrophobicity of the AMP, which in turn affects its antimicrobial and hemolytic activities.^[^ ^27^ ^]^ As another example, the optimal peptide length for MHC class II affinity is ≈18–20 amino acids; peptide elongation beyond this length has no effect or negative impact on affinity.^[^ ^28^ ^]^ If we cannot control the length of the generated sequences well, it is possible to generate more sequences that do not meet the expected requirements due to the stochastic nature of the generation model. Although it is currently possible to impose restrictions on the generated lengths by, for example, constructing classification distributions based on the sequence lengths of short peptide datasets,^[^ ^29^ ^]^ it is not an easy task to fit the corresponding classification distributions better. If the fitting is not good, it is possible that the length of the final sequence obtained is not the length we expect. And most of the generative models currently used to generate peptides are trained on only a single peptide data. In reality, a single peptide may have multiple therapeutic effects. If the generative model can capture the commonality of these different types of peptides, then with only a little guidance to the generative model, high quality peptide sequences of different types can be generated.

In this paper, we propose a Transformer‐based diffusion model for generating therapeutic peptide sequences using pLM embeddings, named CPL‐Diff. Specifically, we employ the ESM‐2 pLM as an encoder to obtain a continuous latent space embedding representation of the protein sequence and introduce an attention masking mechanism in our model so that our model only focuses on the specified portion of the peptide sequence to ensure that the generated peptide sequence is of the specified length. In the sampling phase we use additional peptide category representations to guide our model to generate therapeutic peptides with specified effects. After training, CPL‐Diff can learn the amino acid composition patterns and related physicochemical properties of different peptides. Not only can it generate peptide sequences of specified lengths according to our needs, but also its generated sequences have lower similarity and better physicochemical properties.

We summarize the contributions of our work as follows.

- A diffusion model for the generation of therapeutic peptide sequences using a masking mechanism CPL‐Diff is proposed, which enforces control over the length of the generated peptide sequences without relying on any marginal distribution.
- Use conditional information to guide the generation of therapeutic peptides with different effects.
- Interpretability analysis of CPL‐Diff's ability to control the length of generated sequences. And to quantitatively analyse CPL‐Diff, we predicted the structure of the sequence generated by CPL‐Diff and performed simulated docking experiments.

## 2 Methodology

**Figure 1:**
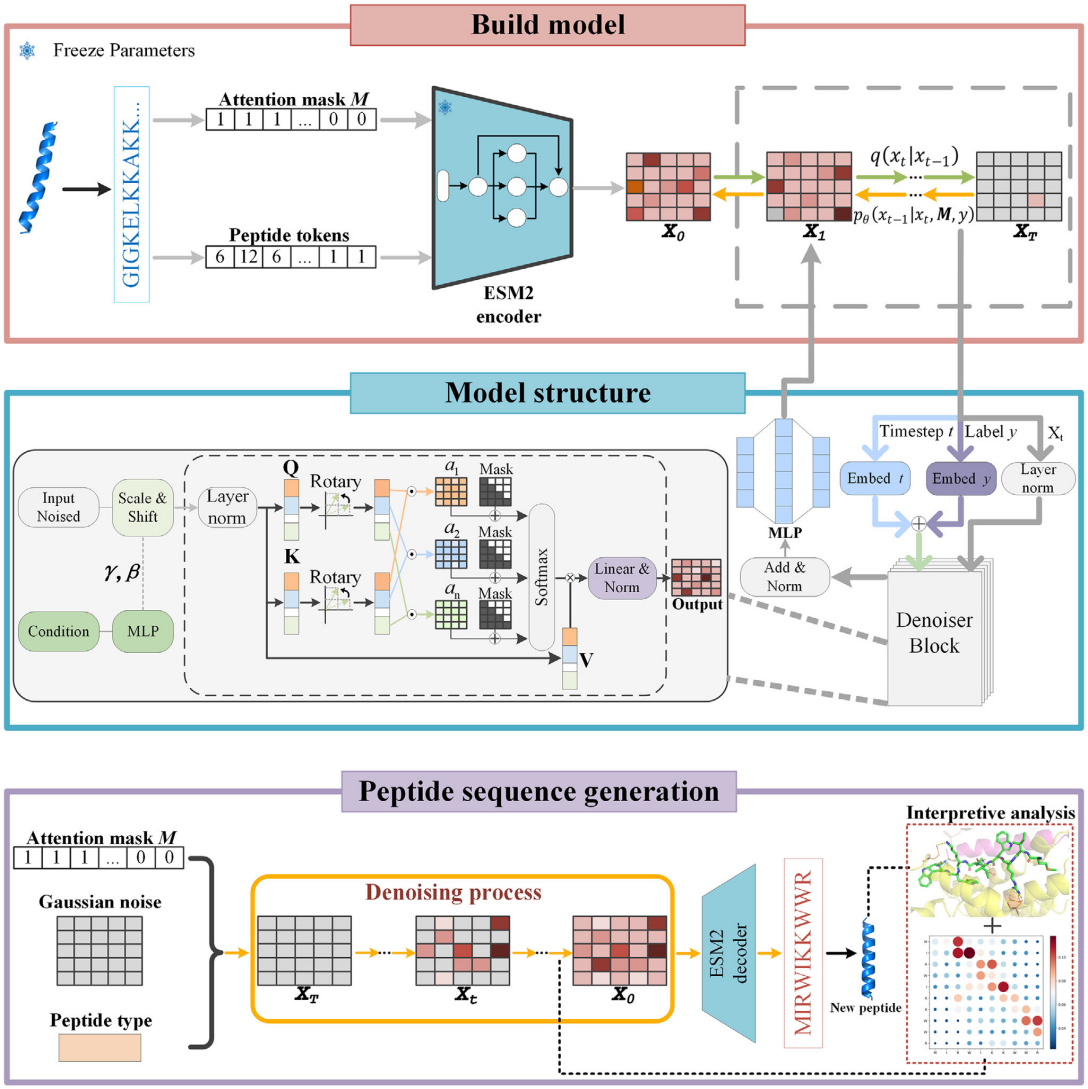
Overview of CPL‐Diff. The red box shows the training process. We first convert the sequences into tokens as well as the corresponding Attention masks. Then, the latent space embeddings of the peptide sequences are extracted using the pre‐trained ESM2. The peptide category labels are probabilistically eliminated for the purpose of combining conditional and unconditional diffusion into one model. In the diffusion process, Gaussian noise is introduced into these latent space embeddings. In the inverse process, the denoiser is trained, and the denoiser is used to reconstruct the noise‐disturbed latent space embeddings. The attention mask and category labels are used as guidance information. The blue box shows the architecture of the denoiser. The denoiser mainly consists of the Multi‐head Attention Layer and the MLP. The denoiser receives four inputs, i.e., the noise‐disturbed latent space embedding, the timestep t, the Attention mask, and the category label y. The purple box shows the sampling process. Sampling starts with pure Gaussian noise, using an Attention mask to control the length of the generated peptide sequence and to provide the type of peptide to be generated. After denoising is complete, the generated latent space embedding is decoded using the ESM2 language model header to obtain a new peptide sequence. Finally, the Attention weight matrix of CPL‐Diff is extracted and combined with simulated docking experiments for interpretability analysis.

In this part, we propose the problem of controlling the length of generated sequences in diffusion models. Subsequently, we will detail the various components of the CPL‐Diff method, including the diffusion model for polypeptide generation, the attention mask control strategy for generating sequence length, and the guided generation strategy. **Figure** **1** is an overview of CPL‐Diff.

### 2.1 Problem Definition

We consider the design of polypeptide sequences as finding a sequence $w\;={\left[w_{i}\right]}_{i=1}^{L}$, where $w_{i}\in \left\{ACDEFGHIKLMNPQRSTVWY\right\}$ is the type of the ith amino acid, and L is the length of the sequence. Our goal is to establish a distribution model for ***w*** based on known polypeptide sequences, and sample from this distribution according to a given sequence length L, ultimately generating a new polypeptide sequence of a specified length.

Peptide sequence design can be achieved by establishing a trained language model *p_lm_* (***w***) from known peptide sequences, and then extracting a new sequence *w* from this model, where *p_lm_* (***w***) is the probability distribution of word sequences. Controllable polypeptide sequence generation refers to the task of extracting a new sequence *w* from a conditional distribution *p*(***w*** |*y*), where *y* represents the control condition.

### 2.2 Advantages of Diffusion Models in Protein Generation

In the Introduction part, we provided a brief overview of the applications of mainstream generative models in sequence generation. In this part, we present a theoretical analysis to elucidate the superiority of diffusion models over other mainstream generative models in the context of protein generation.

Generative models based on RNNs and its variants have been implemented in protein design tasks for both generative and discriminative applications,^[^ ^9^, ^30^, ^31^ ^]^ where protein sequences are generated through autoregressive frameworks. However, these RNN‐based approaches suffer from inherent limitations: short‐term memory constraints and extended gradient propagation paths render them particularly inefficient for long sequence generation, while potential information loss during training may further compromise the quality of the generated sequences.^[^ ^32^ ^]^

The VAE conceptualizes protein sequences as parameterized multivariate distributions,^[^ ^33^, ^34^, ^35^ ^]^ sharing the structural framework of conventional autoencoders but distinguished by probabilistic latent representations of data attributes. In this framework, a decoder generates high‐dimensional novel samples by inputting low‐dimensional data, such as Gaussian noise. However, VAE training is prone to posterior collapse, a phenomenon where the model fails to produce diverse and high‐quality novel samples due to diminished expressiveness in latent space learning.^[^ ^36^ ^]^

GANs constitute an implicit probabilistic model,^[^ ^37^ ^]^ wherein two neural networks—the generator and the discriminator—engage in adversarial competition. Through this framework, the generator learns to synthesize synthetic data that closely resembles the distribution of authentic training samples, while the discriminator provides discriminative feedback to iteratively refine the generator's output. GANs have demonstrated notable efficacy in protein sequence generation tasks.^[^ ^33^, ^38^ ^]^ However, their application is hindered by two critical limitations: gradient convergence instability​and mode collapse. The former impedes learning by decelerating or halting parameter updates, whereas the latter arises when the discriminator fails to differentiate synthetic samples from real ones, driving the generator to produce repetitive, low‐diversity outputs of suboptimal quality.^[^ ^39^ ^]^ Furthermore, maintaining equilibrium between the convergence dynamics of the two networks remains inherently challenging, often leading to training stagnation.^[^ ^40^ ^]^

Compared to the aforementioned generative models, diffusion models exhibit superior performance in three key aspects: ​enhanced sample diversity, ​more stable training dynamics, and ​improved fine‐grained controllability.^[^ ^41^, ^42^ ^]^ Significantly, diffusion models inherently support the generation of proteins with diverse conformations from identical noise inputs—a critical advantage given the dynamic nature of protein structures in biological systems. This intrinsic stochasticity enables a modeling approach that better approximates real‐world protein behavior.^[^ ^32^ ^]^ Consequently, diffusion models represent a more suitable paradigm for protein generation than conventional generative architectures.

### 2.3 Specific Workflow for the CPL‐Diff Network

We will first briefly introduce the diffusion model (DMs).^[^ ^13^ ^]^ DMs is a latent variable model that aims to build a bridge between simple data distributions such as Gaussian distribution and unknown complex data distributions by constructing a Markov chain $x_{0},x_{1},\text{…},x_{T}$, to approximate the unknown complex data distribution *p*(**x**). DMs can be divided into forward and backward processes. The forward process, also known as the diffusion process, is a Markov process that can be represented as $q\;\left(x_{1:T}|x_{0}\right)=\prod_{t=1}^{T}q\left(x_{t}|x_{t-1}\right)$. The Markov process adds Gaussian noise to the original data according to a schedule $\beta_{1},\beta_{2},\text{…},\beta_{T}$:

(1) $$q\left(x_{t}|x_{t-1}\right)=N\left(x_{t};\sqrt{1-\beta_{t}}x_{t-1},\beta_{t}I\right),\;t=1,2,\text{…},T$$

where β_*t*_ controls the amount of noise added at the current timestep *t*. When *t* → ∞, **x**_0_ will be destroyed into pure Gaussian noise.

A significant feature of the forward process is that the state at any timestep t can be directly obtained from **x**_0_:

(2) $$\begin{array}{c} q\;\left(x_{t}|x_{0}\right)=N\left(x_{t};\sqrt{{\bar{\alpha }}_{t}}x_{0},\left(1-{\bar{\alpha }}_{t}\right)I\right) \end{array}$$

where ${\bar{\alpha }}_{t}{=}^{\prime }\prod_{i=1}^{t}\alpha_{i}$ and α_*i*_ =  1 − β_*i*_. For the calculation of each timestep *t*, we first sample ε∼N(0,I) from a Gaussian distribution, and then obtain **x**_***t***_ from **x**_0_ through ${\bar{\alpha }}_{t}$:

(3) $$\begin{array}{c} \;x_{t}=\sqrt{{\bar{\alpha }}_{t}}\;x_{0}+\sqrt{1-{\bar{\alpha }}_{t}}\varepsilon \end{array}$$

Typically, since the schedule β_*t*_ in the forward process *q* is fixed, there are no trainable parameters in the forward process.

The reverse process, also known as the inverse diffusion process, is also a Markov process, which can be represented as $p\;\left(x_{0:T}\right)=p{\left(x_{T}\right)}^{\prime }\prod_{t=1}^{T}p\left(x_{t-1}|x_{t}\right)$. The reverse process starts from Gaussian noise $x_{T}\approx N\left(0,I\right)$ and gradually removes noise to obtain data that has not been corrupted by noise. However, in practical applications, we do not know the specific form of *p*(**x**_***t*** − 1_|**x**_***t***_), but we can define a neural network *p*_θ_ to approximate this distribution, that is, using *p*_θ_(**x**_***t*** − 1_|**x**_***t***_) instead of *p*(**x**_***t*** − 1_|**x**_***t***_) Therefore, the reverse process can be regarded as a parameterized Markov chain:

(4) $$\begin{array}{c} p_{\theta }\;\left(x_{0:T}\right)=p\left(x_{T}\right)\prod_{t=1}^{T}p_{\theta }\left(x_{t-1}|x_{t}\right),\\ p_{\theta }\;\left(x_{t-1}|x_{t}\right)=N\left(x_{t-1};\mu_{\theta }\left(x_{t},t\right),\Sigma_{\theta }\left(x_{t},t\right)\right) \end{array}$$

where *p*_θ_(**x**_0: ***T***_) is the joint probability distribution of $\left(x_{0},x_{1},\text{…},x_{T}\right)$, *p*(**x**_***T***_) is a Gaussian distribution. The mean term µ_θ_(**x**_***t***_,*t*) accepts **x**_***t***_ and *t* as inputs and can be learned. The variance term Σ_θ_(**x**_***t***_,*t*) can be set to β_*t*_ **I** or other lists related to *t* according to the settings of DDPM.

As shown in the red boxed portion of Figure 1, in generating peptides based on latent diffusion, the pre‐trained ESM‐2 encoder is used to map the peptide sequences into a continuous latent space, which is denoted as $x_{0}\in R^{l\times d}$, where l is denoted as the length of the sequences, and *d* is the dimensionality of the latent space embedding. The ESM‐2 encoder is not fine‐tuned throughout the entire process. In the forward process, Gaussian noise is added at each time step. At the same time, the denoiser is trained to reconstruct the noise‐disturbed latent space embedding. The denoiser is trained with the goal of minimizing the l2 loss between predicted **x**_0_ and true **x**_0_.

As shown in the purple boxed portion of Figure 1, peptide sequence sampling starts with pure Gaussian noise and uses Attention mask to indicate the length of the sequence to be generated and specify which type of peptide needs to be generated. The pure Gaussian noise is then progressively denoised by sampling with DDPM. After denoising is complete the obtained latent space embedding is decoded using the ESM2 language modeling header to finally obtain a new peptide sequence with the specified length and type.

In the following part, we will introduce the structure of the denoiser in detail.

#### 2.3.1 Denoiser Module

In the reverse process, we train a neural network to gradually recover the original data by removing noise from the data step by step. Specifically, our denoising network uses the Transformer architecture. The denoiser accepts four inputs: noisy data **x**_***t***_, timestep *t*, Attention mask, and peptide category identity *y*. Where $x_{t}\in R^{l\times d}$. To simplify training, we fix l, which means that all latent space variables obtained after encoding by the ESM2 encoder have the same shape.

To make the sampling process more intuitive, we choose to modify the shape of the attention mask in the denoising process. Specifically, we use **M** to represent the attention mask, and for all elements *m*_*i*,*j*_ of **M**, $m_{i,j}\in \left\{0,1\right\},=1,2,\text{…},batch,j=1,2,\text{…},\max\_len$, where *batch* is the number of sequences to be generated and max_len is the maximum length of the sequence. It should be noted that because ESM2 needs to add special markers at the beginning and end of the sequence, the actual max_len is the true maximum sequence length plus 2. That is to say, in each row vector of **M**, the number of “1”s represents the length of the sequence to be sampled plus 2.

To accelerate the training process and minimize the impact of outliers in the input on model performance during training, we first apply layer normalization to the noisy embedded inputs^[^ ^43^ ^]^:

(5) $$\begin{array}{c} \;{\hat{x}}_{t}^{i}=LayerNorm\;\left({\hat{x}}_{t}^{i}\right)=\gamma \frac{{\hat{x}}_{t}^{i}-E\left[{\hat{x}}_{t}^{i}\right]}{\sqrt{Var\left[{\hat{x}}_{t}^{i}\right]+\varepsilon }}+\beta \end{array}$$

where E[·] represents the mean, *Var*[·] represents the variance, and ***γ*** and ***β*** are learnable parameters used to perform affine transformations on the normalized results. Here, we perform layer normalization on the last dimension of the embedding to ensure that each embedding vector is independently normalized.

Classification‐Guided Generation: We attempted to guide our diffusion model to generate peptides with specified therapeutic effects. Specifically, Dhariwal & Nichol et al.^[^ ^44^ ^]^ introduced a classifier into the diffusion model, at which point the inverse process was modified to include the log‐likelihood gradient of the classifier:

(6) $$\nabla_{x_{t}}logp\;\left(x_{t}|y\right)=\nabla_{x_{t}}\;logp\left(x_{t}\right)+\omega \nabla_{x_{t}}logp\left(y|x_{t}\right)$$

where *p*(*y*|**x**_***t***_) denotes a classifier and ω controls the bootstrap strength. According to Ho & Salimans et al,^[^ ^45^ ^]^ $\nabla_{x_{t}}logp\left(y|x_{t}\right)$ can be further expressed using Bayes' theorem as:

(7) $$\begin{array}{c} \nabla_{x_{t}}\log p\;\left(y|x_{t}\right)=\nabla_{x_{t}}\;\log p\left(x_{t}|y\right)+\nabla_{x_{t}}\log p\left(x_{t}\right),\\ \mathrm{where}\;\nabla_{x_{t}}\log p\;\left(y\right)=0 \end{array}$$

Equation (6) is then obtained by substituting Equation (7):

(8) $$\begin{array}{c} \begin{array}{c} \nabla_{x_{t}}logp\;\left(x_{t}|y\right)=\nabla_{x_{t}}\;logp\left(x_{t}\right)+\omega \left(\nabla_{x_{t}}logp\left(x_{t}|y\right)-\nabla_{x_{t}}logp\left(x_{t}\right)\right)\\ =\left(1-\omega \right)\;\nabla_{x_{t}}logp\left(x_{t}\right)+\omega \nabla_{x_{t}}logp\left(x_{t}|y\right) \end{array} \end{array}$$

We let ω  =  1 + λ, then we have:

(9) $$\nabla_{x_{t}}logp\;\left(x_{t}|y\right)=\left(1+\lambda \right)\;\nabla_{x_{t}}logp\left(x_{t}|y\right)-\lambda \nabla_{x_{t}}logp\left(x_{t}\right)$$

At this point when λ  =   − 1, the first term is 0, at which point the model ignores the given condition. When λ  =  0, the second term is 0, at which point the model attaches conditions. When λ > 0, at this point the model will prioritize conditional generation and move away from the direction of unconditional generation.

We need a conditional and an unconditional network for this process. However, we can treat the unconditional network as a special case of the conditional network, i.e., construct an unconditional logo and replace the other logos with the unconditional logo with some probability during training. In this way, we only need to train one network.

Timestep Encoding: Timestep t is a discrete value. In order to use it as part of the denoising process so that the denoising algorithm knows which timestep of noisy data is currently being processed, we use the sine and cosine position encoding proposed by Vaswani et al.^[^ ^46^ ^]^ to encode the timestep:

(10) $$\begin{array}{c} P\;E_{\left(pos,2i\right)}=\sin\left(\frac{pos}{{10\;000}^{2i/d_{model}}}\right)\;,\\ P\;E_{\left(pos,2i+1\right)}=\cos\left(\frac{pos}{{10\;000}^{2i/d_{model}}}\right) \end{array}$$

where *PE* represents the position encoding, *pos* represents the position, which is the timestep *t*, *d_model_* represents the total dimension after encoding, and i represents the value of the ith dimension in the position encoding matrix, where 0 ≤ *i* ≤ *d_model_* − 1. Sinusoidal position encoding does not introduce additional parameters to the model, thereby reducing training costs.

Conditional Information Embedding: After encoding the timestep, it is embedded as conditional information by summing with the encoded category representation. It is then entered into successive denoising blocks along with the noise‐containing data, the Attention mask with a modified shape. In order for the model to better understand the given conditional information, each denoising block has a separate MLP to further process the encoded conditional information, which is then integrated into the embedding through the feature affine transform operation (FiLM)^[^ ^47^ ^]^:

(11) $$\begin{array}{c} c_{\mathrm{emb}}^{i}=F\left(Silu\left(F\left(PE\left(t\right)\right)\right)\right)+emb\left(y\right),\\ c_{\gamma }^{i},\;c_{\beta }^{i}=chunk\left(c_{\mathrm{emb}}^{i}\right),\\ {\hat{x}}_{t}^{i}=FiLM\;\left({\hat{x}}_{t}^{i-1}\right)=\left(c_{\gamma }^{i}+1\right)\;⊙{\hat{x}}_{t}^{i-1}+c_{\beta }^{i} \end{array}$$

where $c_{\mathrm{emb}}^{i}$ denotes the conditional information embedding of the ith denoising block, F(·) denotes the fully connected layer, *Silu* denotes the activation function, *PE*(·) denotes the positive cosine position encoding function, and *emb*(·) denotes the category identification code. *chunk*(·) denotes the splitting of a 3D tensor into two chunks according to the last dimension. ${\hat{x}}_{t}^{i}$ denotes the input embedding of the ith denoising block, and when *i*  =  0, ${\hat{x}}_{t}^{i}$ denotes the original of the noise‐corrupted data. ⊙ denotes the element multiplication, $c_{\gamma }^{i}$ and $c_{\beta }^{i}$ denote the element scaling and element displacement, and 1 is a tensor of ones to ensure that the scaling factor is centered at one. By using the FiLM layer, the denoiser can better understand the current information about the given conditions and thus perform better denoising.

Attention Layer: After integrating the conditional information into the protein embedding, it will enter the ESM2 attention layer for processing. The attention layer is a multi‐head attention layer. In the attention layer, the input embeddings are first subjected to layer normalization, and then the query, key, and value are computed from the layer‐normalized embeddings:

(12) $$Q^{i}=F_{Q}\;\left({\hat{x}}_{t}^{i}\right),\;K^{i}=F_{K}\;\left({\hat{x}}_{t}^{i}\right),\;V^{i}=F_{V}\;\left({\hat{x}}_{t}^{i}\right)$$

where ∀t∈{1,2,…,T}, **Q** ^**i**^, **K** ^**i**^, and **V** ^**i**^ represent the query, key, and value learned by embedding ${\hat{x}}_{t}^{i}$, respectively. $F_{Q}$, $F_{K}$, and $F_{V}$ represent independent fully connected layers used to learn the query, key, and value from the embedding.

For peptides, the relative positions of amino acids are very important.^[^ ^48^ ^]^ Therefore, in order for our model to understand the relative position information of amino acids in peptide sequences, rotational position encoding (RoPE) is applied to the embedded query and key after calculating them respectively.^[^ ^49^ ^]^ RoPE is a kind of relative position encoding, which realizes the rotational transformations through complex number computation, and integrates the position information into the query and key in the attention layer in the form of multiplication operation, so that the embedded query and keys naturally contain position information. We take query as an example, and for simplicity, we will write **Q** ^**i**^ as ***q***. Specifically, for a 2D vector ***q***, there is the following RoPE expressed in complex numbers:

(13) $$f\;\left(q,m\right)=R_{f}\;\left(q,m\right)\;e^{i⊖f\left(q,m\right)}=qe^{im\theta }$$

where ***q*** represents the vector to be RoPE, *m* represents the position of ***q***, and θ represents the angle of rotation. According to the geometric meaning of complex multiplication, RoPE can be written in the form of matrix multiplication:

(14) $$f\;\left(q,m\right)=\left[\begin{array}{cc} \cos\left(m\theta \right) & -\sin\left(m\theta \right)\\ \sin\left(m\theta \right) & \;\cos\left(m\theta \right) \end{array}\right]\;\cdot \left[\begin{array}{c} q_{0}\\ q_{1} \end{array}\right]$$

where ${\left[\begin{array}{cc} q_{0} & q_{1} \end{array}\right]}^{T}$ represents the vector to be rotated. Since the inner product satisfies linear superposition, for a vector of even dimensionality, RoPE can be represented as:

(15) $$\begin{array}{ccc} & & f\left(q,m\right)=R_{m}\;q\\ & & =\left[\begin{array}{c} q_{0}\\ q_{1}\\ q_{2}\\ q_{3}\\ \vdots \\ q_{d_{model-1}} \end{array}\right]\;⊗\left[\begin{array}{c} \cos\left(m\theta_{0}\right)\\ \cos\left(m\theta_{0}\right)\\ \cos\left(m\theta_{1}\right)\\ \cos\left(m\theta_{1}\right)\\ \vdots \\ \cos\left(m\theta_{d_{model}/2-1}\right) \end{array}\right]+\left[\begin{array}{c} -q_{1}\\ q_{0}\\ -q_{3}\\ q_{2}\\ \vdots \\ q_{d_{model}-1} \end{array}\right]⊗\left[\begin{array}{c} \sin\left(m\theta_{0}\right)\\ \sin\left(m\theta_{0}\right)\\ \sin\left(m\theta_{1}\right)\\ \sin\left(m\theta_{1}\right)\\ \vdots \\ \sin\left(m\theta_{d_{model}/2-1}\right) \end{array}\right],\\ & & \mathrm{where}\;R_{m}\\ & & =\left[\begin{array}{cccccc} \cos\left(m\theta_{0}\right) & -\sin\left(m\theta_{0}\right) & 0 & \text{…} & 0 & 0\\ \sin\left(m\theta_{0}\right) & \cos\left(m\theta_{0}\right) & 0 & \text{…} & 0 & 0\\ 0 & 0 & \cos\left(m\theta_{1}\right) & \text{…} & 0 & 0\\ 0 & 0 & \sin\left(m\theta_{1}\right) & \text{…} & 0 & 0\\ \vdots & \vdots & \vdots & \ddots & \vdots & \vdots \\ 0 & 0 & 0 & \text{…} & \cos\left(m\theta_{d_{model}/2-1}\right) & -\sin\left(m\theta_{d_{model}/2-1}\right)\\ 0 & 0 & 0 & \text{…} & \sin\left(m\theta_{d_{model}/2-1}\right) & \cos\left(m\theta_{d_{model}/2-1}\right) \end{array}\right]\;\;\;\; \end{array}$$

where $R_{m}$ represents the rotation matrix at position m, and since $R_{m}$ is an orthogonal matrix, it does not change the stability of the model. ⊗ represents bitwise multiplication. Here, the value of θ_i_ is based on the Sinusoidal position encoding scheme,^[^ ^46^ ^]^ which is $\theta_{i}=\frac{\mathrm{pos}}{{10\;000}^{-2i/d_{\mathrm{model}}}}$, where i represents the value of the ith dimension in the position encoding matrix(0 ≤ i ≤ d_model_ − 1), pos represents the position, and d_model_ represents the total dimension after encoding. In practical applications, we can perform the following operations to obtain the rotated query and key. Take query as an example. First, split the query into $Q_{1}^{i}$ and $Q_{2}^{i}$ based on the last dimension, then take the opposite number of $Q_{2}^{i}$ and merge them again:

(16) $$\begin{array}{c} Q_{1}^{i},\;Q_{2}^{i}=chunk\left(Q^{i}\right),\\ {\hat{Q}}^{i}=concat\left(-Q_{2}^{i},Q_{1}^{i}\right) \end{array}$$

The position list ***P*** is an arithmetic sequence with a minimum value of 1, a maximum value of L−1, and a common difference of 1. Perform outer product operation on the position list ***P*** and the argument list ***θ***, and perform cosine and sine operations on each element in the resulting matrix:

(17) $$\begin{array}{c} r=P⊗\theta ,\\ \;r_{\cos}=\cos\left(r\right)\;,\;r_{\sin}=\sin\left(r\right) \end{array}$$

Finally, perform a rotation operation to obtain the rotated **Q** ^**i**^:

(18) $${\tilde{Q}}^{i}=Q^{i}\;r_{\cos}+{\hat{Q}}^{i}r_{\sin}$$

For **K** ^**i**^, we only need to perform the same operation as obtaining ${\tilde{Q}}^{i}$ to get the rotated **K** ^**i**^, denoted as ${\tilde{K}}^{i}$. Then, perform the dot product operation between ${\tilde{Q}}^{i}$ and **K** ^**i**^:

(19) $$\psi^{i}={\tilde{Q}}^{i}\;{\left({\tilde{K}}^{i}\right)}^{T}$$

In order to let the denoiser know the sequence length corresponding to the currently processed ${\hat{x}}_{t}^{i}$ after calculating **ψ** ^**i**^, a mask matrix is added:

(20) $$\;\psi^{i}=\psi^{i}+M$$

where $M\in R^{\left(max\_len+2)\times (max\_len+2\right)}$. The first *L* + 2 columns (1≤L≤max_len) of each row of ***M*** are 1, and the rest of the columns are 0. The reason for adding 2 is because we want the model to handle the two special tokens CLS and EOS as well. This setup not only allows the model to focus more on the effective parts, but also allows us to have good interpretability of the model.

Then calculate the attention of the ith denoising block:

(21) $$Att\;\left(\psi^{i},V^{i}\right)=softmax\left(\psi^{i}\right)V^{i}$$

Furthermore, based on the extension in,^[^ ^46^ ^]^ we can obtain the multi‐head attention of the i‐th denoising block, which is calculated as follows:

(22) $$\begin{array}{c} MulitHead\left(Q^{i},k^{i},V^{i}\right)=F_{M}\left({\left[\mathrm{hea}d_{m}\right]}_{m=1}^{n}\right),\\ \mathrm{hea}\;d_{m}=Att_{m}\left(\psi^{i},V^{i}\right) \end{array}$$

where $F_{M}$ is a fully connected layer connecting the n heads. Finally, the output embedding is obtained through a feed‐forward neural network and layer normalization.

After ${\hat{x}}_{t}^{0}$ is processed through multiple consecutive denoising blocks, its output is connected to **x**_***t***_ for residual connection and layer normalization, and finally passes through an MLP to obtain the predicted polypeptide sequence embedding ${\hat{x}}_{0}$ that is not corrupted by noise.

#### 2.3.2 Training Phase

After a detailed introduction to the forward diffusion and reverse denoising processes, we will proceed to explain how to train the diffusion model. For Σ_θ_ in Equation 4, we adopt a fixed list based on the conclusion of DDPM, namely $\Sigma_{t}=\frac{1-{\bar{\alpha }}_{t-1}}{1-{\bar{\alpha }}_{t}}\;\beta_{t}$. where ${\bar{\alpha }}_{t}$ depends on the noise plan adopted by the diffusion model. Here, our noise plan adopts the sqrt noise plan proposed by Diffusion‐LM^[^ ^50^ ^]^:

(23) $${\bar{\alpha }}_{t}=1-\sqrt{\frac{t}{T+S}}$$

where *S* is a smaller constant. Then, according to ${\bar{\alpha }}_{t}{=}^{\prime }\prod_{i=1}^{t}\alpha_{i}\;,\;\alpha_{i}=1-\beta_{i}$, the β_*t*_ list can be recursively calculated.

For µ_θ_ in Equation 4, we can achieve it by training a neural network ${\hat{z}}_{\theta }$. According to the conclusion drawn by Diffusion‐LM, for the text latent variable diffusion model, the neural network trained to directly predict the embedding without noise will perform better than predicting the effect of noise. Therefore, our denoising model directly predicts the original embedding **x**_0_. Therefore, the optimization objective of the model is to minimize the l2 loss between the predicted *x*_0_ and the true *x*_0_:

(24) $$L\;\left(\theta \right)=E_{x_{0}∼p_{data},t∼U\left(1,T\right)}\;\left[\left|\right|{\hat{z}}_{\theta }\left(x_{t},t,M\right)-x_{0}{\left|\right|}_{2}^{2}\right]$$

where **M** represents the mask matrix, which represents the length of the sequence corresponding to the latent space embedding **x**_0_. It should be noted that, since ESM2 needs to add <CLS> and <EOS> tokens at the beginning and end of the sequence, that is, $M\in R^{batch\times (\max\_len+2)},R_{i,j}\in \left\{0,1\right\}$.

We have chosen ESM2‐8 M as our pLM. During use, separate the encoding and decoding parts of the ESM2‐8M. The parameters of the encoding part will not be adjusted during the entire training process, while the decoding part will use the collected polypeptide sequences for fine‐tuning.

Algorithm 1 shows the training process of the denoiser. The peptide sequence ***w*** and the corresponding peptide type identifier *y* are first extracted from the training set. Then with a certain probability, some of the marks are turned into unclassified marks. Then, the vectorized sequence and the corresponding mask are used to obtain the ESM2 latent space embedding **x**_0_ corresponding to the sequence. Then, we randomly sample a timestep 𝑡 from the set of integers {1,…,T} and sample a random noise ε from a Gaussian distribution. Then, the denoising unit ${\hat{z}}_{\theta }$ is optimized using the gradient descent strategy. Repeat the above process until ${\hat{z}}_{\theta }$ converges.

Algorithm 1 **Diffusion Training**

**Table:** 

Require: Peptide sequences ***w***, Peptide embeddings D, Original data sample **x**_0_, Latent variables $x_{1},\text{…},x_{T}$, Protein sequence encoder *E*(), Tokenizer *T*(), Sequence ids seq_ids, Attention mask **M**, Peptide type identifier *y*, Denoising model ${\hat{z}}_{\theta }$, Timestep T, Noise ε, Noise schedule ${\bar{\alpha }}_{t}$, Learning rate η		
1:	**repeat**	
2:	(w,y)∼p(w,y)	Sample sequence with condition from dataset
3:	*y* ← ∅* *with probability *p_uncond_*	Randomly selected identifiers become unconditionally
4:	seq_ids, **M =** *T*(***w***)	Get sequence ids and Attention mask
5:	$\;x_{0}=E\left(\mathrm{seq}\_\mathrm{ids},\;M\right)\;\approx \;D$	Encode sampled data
6:	t∼U({1,…,T})	Sample timestep
7:	ε∼N(0,I)	Sample noise
8:	$\;x_{t}=\sqrt{{\bar{\alpha }}_{t}}\;x_{0}+\sqrt{1-{\bar{\alpha }}_{t}}\varepsilon$	Compute latent variables
9:	$\;L=\left|\right|{\hat{z}}_{\theta }\left(x_{t},\;t,M,y\right)-x_{0}{\left|\right|}_{2}^{2}$	Compute loss
10:	$\;\theta =\theta -\eta \nabla_{\theta }L$	Update parameters
11:	**until** converged

#### 2.3.3 Peptide Sequence Sampling

Algorithm 2 shows the sampling process of our model. The sampling method follows the DDPM approach, where noise‐free embeddings are eventually obtained through stepwise denoising. The denoising will predict both the conditional noise‐free embedding ${\tilde{x}}_{0,y}$ and the unconditional noise‐free embedding ${\tilde{x}}_{0,\emptyset }$. Then ${\tilde{x}}_{0}$ is obtained by a linear combination of ${\tilde{x}}_{0,y}$ and ${\tilde{x}}_{0,\emptyset }$ and the degree of bias toward the specified polypeptide type is controlled by the bootstrap strength λ. The mean ${\tilde{\mu }}_{t}$ of $q(x_{t-1}|x_{1},{\tilde{x}}_{0})$ is then computed from the obtained ${\tilde{x}}_{0}$, and for simplicity we fix the variance. Finally, **x**_***t*** − 1_ is obtained by the reparameterization trick.

Algorithm 2 **Diffusion Sampling**

**Table:** 

Require: Estimated original data ${\tilde{x}}_{0}$, Latent variables $x_{1},\text{…},x_{T}$, Denoising model ${\hat{z}}_{\theta }$, Timestep *T*, Noise ε, Noise schedule ${\bar{\alpha }}_{t}=\prod_{i=1}^{t}\alpha_{i}$, Noise intensity β_*t*_ = 1 − α_*t*_, Peptide type identifier *y*, Sample length L, Attention mask **M**, Gradient scale λ, Protein sequence decoder *D*()		
1:	$\;x_{T}\approx N\left(0,I\right)$	Sample the initial latent variable
2:	L → **M**	Generate an attention mask by specifying a length
3:	**for** t = T,…,1 **do**	
4:	$\;{\tilde{x}}_{0,y}={\hat{z}}_{\theta }\;\left(x_{t},t,M,y\right)$	Estimate original data **x**_0_ with condition
5:	$\;{\tilde{x}}_{0,\emptyset }={\hat{z}}_{\theta }\;\left(x_{t},t,M\right)$	Estimate original data **x**_0_ with uncondition
6:	$\;{\tilde{x}}_{0}=\left(1+\lambda \right)\;{\tilde{x}}_{0,y}-\lambda {\tilde{x}}_{0,\emptyset }$	
4:	**if** t = 1 **then**	
5:	**return** ${\tilde{x}}_{0}$	Get generated data
6:	$\;{\tilde{\mu }}_{t}=\frac{\sqrt{{\bar{\alpha }}_{t-1}}\beta_{t}}{1-{\bar{\alpha }}_{t}}\;{\tilde{x}}_{0}+\frac{\sqrt{{\bar{\alpha }}_{t}}\left(1-{\bar{\alpha }}_{t-1}\right)}{1-{\bar{\alpha }}_{t}}x_{t}$	Compute mean
7:	$\;{\tilde{\Sigma }}_{t}=\frac{1-{\bar{\alpha }}_{t-1}}{1-{\bar{\alpha }}_{t}}\;\beta_{t}\cdot 1_{\left(max\_len,\;320\right)}$	Compute variance
8:	ε≈N(0,I)	Sample noise
9:	$\;x_{t-1}={\tilde{\mu }}_{t}+exp\left(0.5\times \log({\tilde{\Sigma }}_{t})\right)\varepsilon$	Compute the next latent variable
10:	**end for**	
11:	$\;\hat{w}=D\left({\tilde{x}}_{0}\right)$	Decode generated data

#### 2.3.4 Sequence Decode

After obtaining the generated ESM2 latent space embeddings, we decode them using the language modeling header that comes with ESM2. The decoder consists of two linear layers and an activation function; the ESM2 latent space embeddings are processed by the decoder, and the LogSoftMax activation function is used to obtain the logarithm of the probability of each vocabulary word, and the one with the largest value is selected as the word at the current position. After determining all the output words, a sequence is considered valid if the first word is labeled CLS and the word not in the second position is labeled EOS. Finally, we select the content between the CLS and EOS tags as the final generated peptide sequence; the CLS tags, EOS tags, and the rest are discarded.

## 3 Experiments

In this part, we first present the experimental dataset, evaluation metrics, and implementation details, and then conduct comprehensive experiments on the dataset and compare it with other existing methods. Finally, we conduct an ablation study to analyze the impact on model performance when using masks to control the length of the generated sequences in our framework.

### 3.1 Experimental Datasets

We collected peptide sequences from the following publicly available databases: APD3,^[^ ^51^ ^]^ CAMPR4,^[^ ^52^ ^]^ dbAMP2,^[^ ^53^ ^]^ LAMP2,^[^ ^54^ ^]^ DRAMP 3.0^[^ ^55^ ^]^ DBAASP v3,^[^ ^56^ ^]^ and GRAMPA.^[^ ^57^ ^]^ We collected peptide sequences labeled antibacterial, antifungal, antiviral, and antimicrobial from the above databases. For the dataset used to train the denoising model, the data were screened according to the following criteria: 1) sequence length was limited to 5 to 50 amino acids; 2) only uppercase letters were included, and “U, Z, O, B, J” residues were excluded; and 3) sequences containing “X” were excluded. After screening, the sequences were merged and, duplicates were removed, resulting in 23465 usable peptide sequences. We grouped antibacterial and antimicrobial into antimicrobial and divided the dataset into training and testing sets in a ratio of 8:2. The distribution of each category in the dataset is shown in **Table** **1**. All data were collected up to June 12, 2024.

**Table 1:** Distribution of each category in the dataset.

	Benchmark	Independent
Antimicrobial	11 347	2837
Antifungal	4658	1165
Antiviral	2767	691
Total	18 772	4693

### 3.2 Baseline Models

We compare our CPL‐Diff with the following existing methods. For each method described below, we train on the same dataset unless otherwise noted. Each method is trained using either optimal or default hyperparameters. We only consider methods for which the source code is publicly available.

LSTM‐RNN,^[^ ^9^ ^]^ an RNN‐based model for capturing patterns in peptide sequence data, which generates new peptide sequences by autoregression.

AMPGAN^[^ ^33^ ^]^ encodes peptide sequences using the PC6 protein coding method and generates new peptide sequences from the distribution of the base peptide using a GAN.

HydrAMP,^[^ ^11^ ^]^ a CVAE‐based model. The model comes with a MIC classifier for AMP that learns the hidden space of biologically significant peptides to generate AMP sequences without constraints. Since the method relies on specific biological knowledge, we directly used the generated sequences provided by HydrAMP.

ProGen,^[^ ^58^ ^]^ a Transformer‐based protein sequence generation model, employs an autoregressive approach for sequence synthesis while enabling function‐oriented protein design through conditional label integration.

### 3.3 Evaluation Metrics

ESM‐2 Pseudo‐Perplexity: We use the ESM2 pseudo‐complexity^[^ ^21^ ^]^ to assess the generation quality of generative models. In general, a lower value of pseudo‐complexity indicates a higher confidence level. Specifically, the pseudo‐complexity is computed as an exponent of the negative pseudo‐logarithmic probability of a sequence. This index generates a deterministic value for each sequence, and the calculation requires L positive passes, where L represents the length of the input sequence. The calculation is done as follows:

(25) $$PPL\;\left(w\right)=\exp\left\{-\frac{1}{L}\sum_{i=1}^{L}logp\left(w_{i}|w_{j\neq i}\right)\right\}$$

Predicted Local‐Distance Difference Test (pLDDT)^[^ ^59^ ^]^: This metric is a common metric used in the prediction of protein structures. Briefly, pLDDT evaluates the difference in distance between the position of each residue in the predicted protein structure and the actual structure. A lower score indicates a larger difference between the predicted and actual structure. We used ESMfold to predict the 3D structure of a given peptide sequence. For each amino acid in the predicted structure, ESMfold provides a pLDDT score. We take the average of the confidence scores for all amino acids as the overall confidence score for the peptide.

Instability: The instability score is a measure of peptide stability based on the amino acid composition of the generated sequence.^[^ ^60^ ^]^ A lower score indicates that the peptide is more likely to remain stable. We used the peptide descriptors in the modlAMP package^[^ ^61^ ^]^ to assess the instability score.

Similarity: The similarity score evaluates the comparison score between the generated peptide sequence and the existing sequences in the corresponding peptide dataset. A lower comparison score indicates that the generated peptide sequence is more novel. We calculated this using the PairwiseAligner and BLOSUM62 pairwise scoring matrices^[^ ^62^ ^]^ in the biopython package.^[^ ^63^ ^]^

External Classifier: We use an external classifier to evaluate the proportion of the generated sequences with the specified treatment effect, with the obtained proportions all denoted as “Activity”. The activity prediction of AMP was performed using the Random Forest (RF) AMP activity classifier on CAMPR4.^[^ ^52^ ^]^ The activity prediction of AFP was performed using the AFP activity classifier on Antifungipept.^[^ ^64^ ^]^ The activity prediction of AVP was obtained using the AVP activity classifier on Stack‐AVP.^[^ ^65^ ^]^

Physicochemical Properties: We compared the peptides generated by CPL‐Diff with those generated by other models and with real peptide sequences. For the assessment of physicochemical properties, we chose charge, isoelectric point, hydrophobicity, and aromaticity to evaluate the peptide sequences generated by each model. The isoelectric point refers to the solution pH at which the net charge of all basic (positively charged) and acidic (negatively charged) amino acids in the peptide molecule is zero. Charge calculations were performed according to Bjellqvist's method,^[^ ^66^ ^]^ which assesses the net charge at different pH conditions. Hydrophobicity was quantified using the Eisenberg scale,^[^ ^67^ ^]^ which is a measure of structural amphiphilicity of peptides. Aromaticity was assessed based on the occurrence of phenylalanine, tryptophan, and tyrosine.^[^ ^68^ ^]^ All the physicochemical properties mentioned were calculated using the modlAMP toolkit.^[^ ^61^ ^]^ Where the pH for calculating charge was set to 7.4 and the window size for calculating hydrophobicity was set to 7 as suggested by Eisenberg et al.

### 3.4 Implementation Details

Our model is trained on a single NVIDIA Tesla A100 80G GPU. The batch size is set to 64. The initial learning rate was set to 10^−8^ and boosted to 9.84 × 10^−4^ within 10 000 batches by cosine learning rate warm‐up. This is followed by a cosine learning rate decay scheme to 10^−5^ within 200 000 batches and subsequently fixing that learning rate. The model loss was calculated using l2 loss between the predicted original embedding and the true original embedding. We used an exponential moving average (EMA) with a decay coefficient of 0.99 to smooth the entire training process. The entire implementation uses PyTorch 1.13.1 and Python 3.8.18.

We use the sqrt noise schedule proposed by Diffusion‐LM, where the constant *S* is set to 10^−4^. The diffusion time step is set to 2000. We use a 6‐layer Attention Layer with 20 attention heads and 320 hidden sizes as the backbone of the diffusion model. The probability of eliminating polypeptide category identification is set to 0.1. For detailed information regarding the selection of hyperparameters, please refer to the Part S1 (Supporting Information).

### 3.5 Results

#### 3.5.1 Comparison with Baseline Model

We let all models (our model and the baseline model) generate 10 000 AMP samples, 5000 AFP samples, and 5000 AVP samples. **Table** **2**, **Table** **3**, and **Table** **4** show the performance comparison between CPL‐Diff and the baseline model in terms of generating AMP, AFP, and AVP, respectively. It is important to note that our model allows for the control of the length of generated peptide sequences. To simulate the randomness present in other models, we chose to sample the length of the generated sequences from a uniform distribution during the sampling process. Since we adopted a classifier‐free guided diffusion model, different guiding strengths will result in different sampling outcomes. Therefore, in this part, we set the guiding strengths for AMP, AFP, and AVP to 1.5, 1.5, and 1.0, respectively. For the results of other guiding strengths, please refer to Tables S8–S10, and Figure S1 (Supporting Information).

**Table 2:** Performance evaluation of model generation of AMPs.“↑” indicates that the higher the metric, the better. “↓”indicates that the lower the metric, the better. Boldface indicates the best performance.

Model	Perplexity↓	pLDDT↑	Instability↓	Similarity↓	Activity↑
Train/Real AMPs	15.3703	70.5004	41.6195	–	–
Random sequences	23.8206	56.8559	43.5227	36.9055	0.1382
LSTM‐RNN	17.8742	65.1393	41.4064	34.3996	0.7049
AMP‐GAN	17.1979	66.6217	42.0839	32.5901	0.8508
HydrAMP	13.9181	66.3648	75.0801	35.5401	0.8450
ProGen	15.8686	67.0492	40.7964	36.4182	0.7261
CPL‐Diff	**10.8500**	**67.3976**	**39.3651**	**32.4198**	**0.9631**

**Table 3:** Performance evaluation of model generation of AFPs.“↑” indicates that the higher the metric, the better. “↓”indicates that the lower the metric, the better. Boldface indicates the best performance.

Model	Perplexity↓	pLDDT↑	Instability↓	Similarity↓	Activity↑
Train/Real AFPs	14.6803	71.5134	41.5572	–	–
Random sequences	23.7935	56.7997	43.7434	36.7187	0.1810
LSTM‐RNN	17.2989	65.5043	42.3427	33.2955	0.7032
AMP‐GAN	17.0203	66.9977	40.4288	32.7803	0.7514
ProGen	15.6410	62.7835	39.9445	**32.4573**	0.8174
CPL‐Diff	**10.5346**	**70.1241**	**33.2625**	33.0907	**0.8822**

**Table 4:** Performance evaluation of model generation of AVPs.“↑” indicates that the higher the metric, the better. “↓”indicates that the lower the metric, the better. Boldface indicates the best performance.

Model	Perplexity↓	pLDDT↑	Instability↓	Similarity↓	Activity↑
Train/Real AVPs	17.7406	70.1789	46.0613	–	–
Random sequences	23.7893	56.6697	44.0295	36.3364	0.1582
LSTM‐RNN	19.6750	65.9239	**43.2938**	29.4890	0.6770
AMP‐GAN	18.9127	67.5462	43.9076	29.0361	0.7150
ProGen	17.5505	65.6670	49.5000	31.3414	0.6626
CPL‐Diff	**13.4048**	**70.0062**	44.4324	**27.3311**	**0.7596**

Compared to all baselines, the three peptides generated by CPL‐Diff outperform the baseline model in most metrics. In particular, in terms of perplexity, our model is lower than all baseline models, indicating that CPL‐Diff generates peptide sequences with higher confidence. Furthermore, the activity levels of the three types of peptide sequences generated by CPL‐Diff surpass those of all baseline models, indicating that CPL‐Diff has a greater potential for generating active peptide sequences compared to the baseline models. Specifically for each peptide, CPL‐Diff outperforms all baseline models in generating AMP sequences. We also note that the AMP sequences generated by CPL‐Diff exhibit higher stability than real AMPs. For generating AFP sequences, CPL‐Diff is optimal in all metrics except similarity scores. For generating AVP sequences, CPL‐Diff is optimal in all metrics except instability scores. The reason why some of the metrics are sub‐optimal in terms of generating AFP sequences and generating AVP sequences may be due to the fact that the number of peptide sequences in each category of our dataset is imbalanced as shown in Part 3.1, which leads to the fact that our model doesn't learn the features of peptides with a smaller number of peptides very well. But despite this, our model outperforms the limiting model in most of the metrics. And it can be seen that CPL‐Diff can still generate more novel peptide sequences even when the length of the sequences generated by CPL‐Diff is specified. This also confirms our earlier statement that training with different types of peptides helps the model capture peptide commonalities.

**Figure 2:**
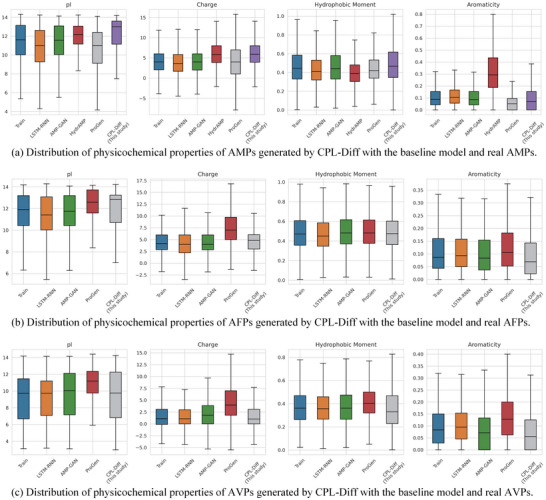
Distribution of physicochemical properties (including isoelectric point (pI), charge, hydrophobicity ratio, and aromaticity) of peptides generated by CPL‐Diff and baseline models and real peptides. a) Distribution of physicochemical properties of AMPs and real AMPs generated by CPL‐Diff and baseline models (including LSTM‐RNN, AMP‐GAN, HydrAMP, and ProGen). The number of samples generated is 10 000. b) Distribution of physicochemical properties of AFPs and real AFPs generated by CPL‐Diff and baseline models (including LSTM‐RNN, AMP‐GAN, and ProGen). The number of samples generated is 5000. c) Distribution of physicochemical properties of AVPs and real AVPs generated by CPL‐Diff and baseline models (including LSTM‐RNN, AMP‐GAN, and ProGen). The number of samples generated is 5000.

The analysis of the physicochemical properties of AMPs is shown in **Figure** **2**a. CPL‐Diff generated AMPs have pI and charges that are very close in distribution to real AMPs. CPL‐Diff and HydrAMP both exhibit higher pI and charge. The variance in charge for ProGen was notably extensive. However, CPL‐Diff demonstrated a higher upper quartile of pI, alongside a lower quartile that surpasses that of real AMPs. This suggests that CPL‐Diff can generate AMPs with potentially biologically active advantages. In terms of global hydrophobic moments and aromaticity, the distribution of the models is closer to that of real AMPs, except for HydrAMP. The reason why HydrAMP exhibits a very high aromaticity may be due to the fact that it frequently adds aromatic amino acids to the peptide, at the cost of a greater reduction of its hydrophobicity, which would somewhat reduce its hydrophobicity. This reduces the AMP activity to a certain extent. Moreover, we can note that the AMP generated by CPL‐Diff exhibits a wider interquartile range of hydrophobicity and aromaticity. This broadening of the range may lead to the generation of novel AMPs with unique properties that can be applied to a variety of therapeutic applications.

The analysis of the physicochemical properties of AFPs is shown in Figure 2b. CPL‐Diff The distribution of pI and charge of the generated AFPs is also very close to that of real AFPs. In contrast, the charge distribution of AFPs produced by ProGen deviates significantly from that of real AFPs. Furthermore, CPL‐Diff also exhibits a higher upper quartile compared to real AFPs. This suggests that CPL‐Diff can generate AFPs with potential bioactive advantages. in terms of global hydrophobic moments and aromaticity, all baseline models are close to the distribution of real AFPs in terms of distribution. It can be noted that CPL‐Diff has a higher lower quartile in hydrophobicity than real AFPs, suggesting that CPL‐Diff is relatively less likely to be able to generate less hydrophobic AFPs. And CPL‐Diff has a wider range in aromaticity. This suggests that CPL‐Diff also has the potential to generate novel AFPs with unique characteristics.

The analysis of the physicochemical properties of AVPs is shown in Figure 2c. The charge distribution of AVPs generated by ProGen also shows a significant deviation from that of real AVPs. The distribution of pI and charges of AVPs generated by CPL‐Diff closely resembles that of authentic AVPs. Moreover, the pI values generated by CPL‐Diff exhibit a higher upper quartile and maximum value compared to those of real AVPs. This suggests that CPL‐Diff can still generate peptides with higher activity than real AVPs. In terms of global hydrophobic moment, CPL‐Diff likewise exhibits a higher maximum point. In terms of aromaticity, CPL‐Diff also a wider range. This suggests that CPL‐Diff also has the ability to produce new AVPs with unique properties.

We further conducted an in‐depth analysis of the generated polypeptide sequences across different lengths. For each baseline model, we randomly sampled 20 sequences from each length category of the generated polypeptide sequences. Similarly, CPL‐Diff was tasked to generate 20 polypeptide sequences for each sequence length. It is important to note that the maximum sequence length provided by the HydrAMP team is 25.

**Figure 3:**
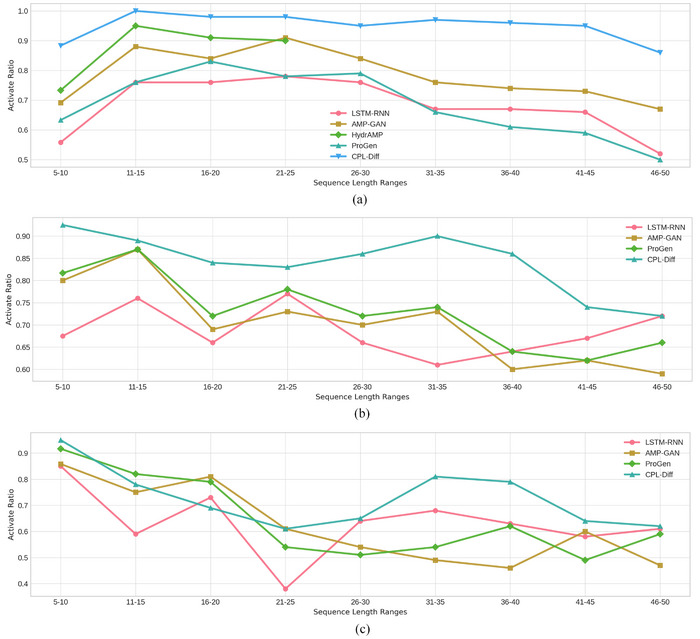
Variation in the proportion of active peptides across different length categories generated by CPL‐Diff with the baseline models. The x‐axis of each subplot represents the sequence length categories, while the y‐axis represents the proportion of peptide activity. The number of peptide sequences for each length is consistent across all models (*n* = 20). a) Variation in the proportion of active AMPs across different length categories generated by CPL‐Diff and baseline models, with the maximum sequence length for HydrAMP being 25. b) Variation in the proportion of active AFPs across different length categories generated by CPL‐Diff and baseline models. c) Variation in the proportion of active AVPs across different length categories generated by CPL‐Diff and baseline models.

The proportion of active polypeptide sequences of varying lengths generated by each model is illustrated in **Figure** **3**. It can be observed that, for the three types of peptides, the trend in activity variation across different length categories for all models closely mirrors the trend in the proportion of different lengths within the peptide dataset (Figure S2, Supporting Information). Moreover, the trend in activity variation for CPL‐Diff is notably smoother compared to other baseline models. Particularly for AMPs, by controlling the generated sequence length, even when there is a limited amount of longer short peptides data, the trend in activity variation remains relatively smooth when generating longer polypeptides. Although the trend in activity variation for AVPs generated by CPL‐Diff is not as smooth as that of AMPs and AFPs generated by CPL‐Diff, it is still generally smoother than that of the baseline models. This indicates that CPL‐Diff can leverage additional length control information and category‐specific information to facilitate further learning of the characteristics of the polypeptide sequence space corresponding to specific lengths. Consequently, this mitigates the issue of a restricted exploration space during generation due to insufficient training data.

Taken together, all the baseline models can only generate one type of peptide sequence, and all of them are trained using only one type of peptide dataset, and thus do not show significant advantages over real peptides in some performance metrics and physicochemical properties. On the other hand, our CPL‐Diff is trained with three types of peptide sequences, which allows us to capture the commonality of peptides and thus has the ability to generate new peptides with significant advantages over real peptides. And since we used a diffusion model without classifier guidance (Part 2.2.1), we can allow CPL‐Diff to choose one of the three types for generation. This can greatly reduce the cost of training. Although the imbalance of the dataset causes some of the peptides generated by CPL‐Diff to be inferior in some metrics. However, taken together, CPL‐Diff still has the potential to generate highly active peptides. It should be noted that since CPL‐Diff can specify the length of the generated peptide sequences, the novelty of the generated peptide sequences can still be guaranteed in this case, which is sufficient to show that our method is advantageous.

#### 3.5.2 Effectiveness of CPL‐Diff's Learning Competencies

##### Amino Acid Composition Analysis

**Figure 4:**
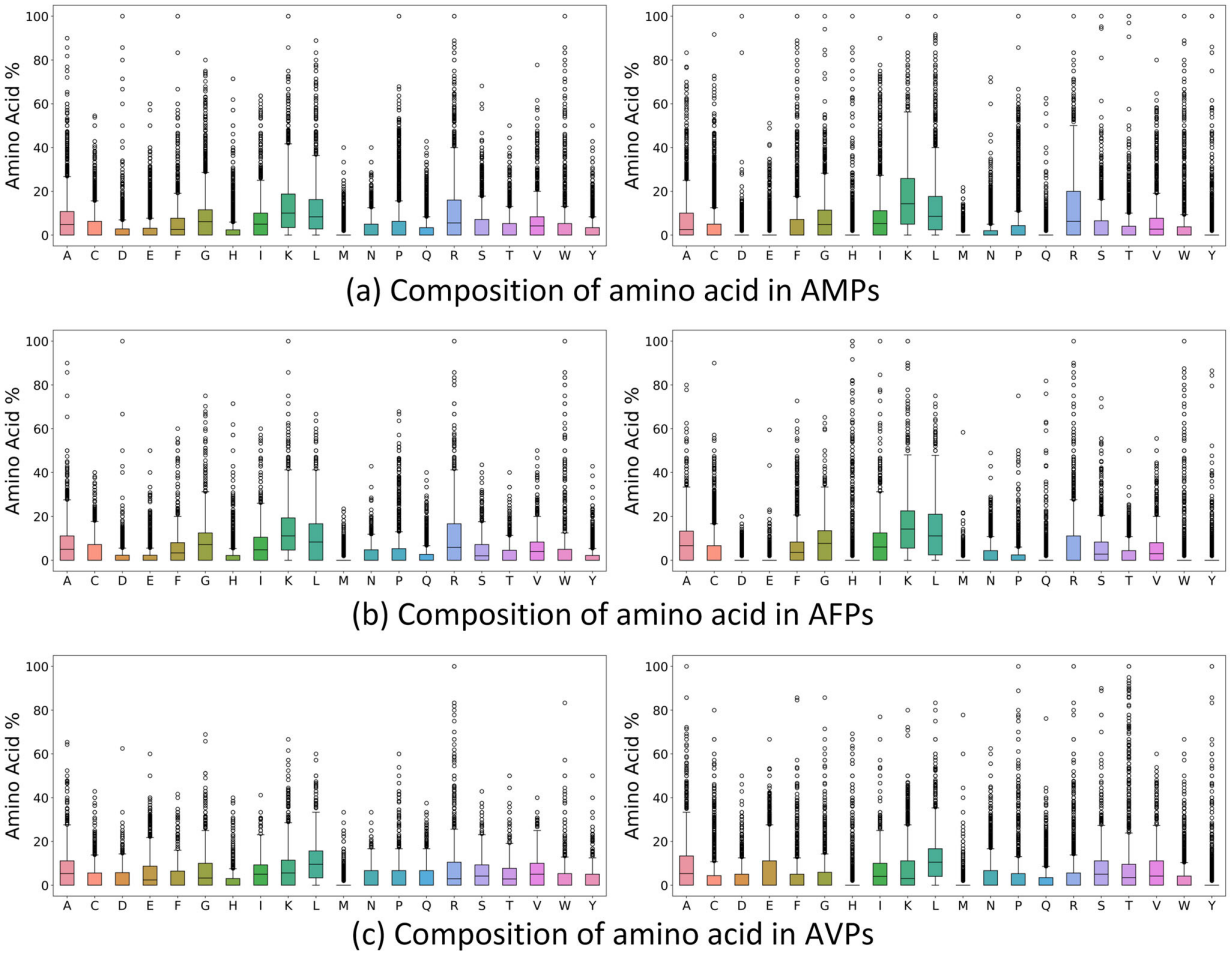
Distribution of amino acid occurrence frequencies. a) Amino acid occurrence frequencies of AMPs. Frequency of amino acid occurrence for real AMPs on the left and amino acid occurrence for CPL‐Diff‐generated AMPs on the right. b) Frequency of amino acid occurrence for AFPs. Frequency of amino acid occurrence for real AFPs on the left and amino acid occurrence for CPL‐Diff‐generated AFPs on the right. c) Frequency of amino acid occurrence for AVPs. Frequency of amino acid occurrence of real AVPs on the left and amino acid occurrence of CPL‐Diff‐generated AVPs on the right.

To further evaluate CPL‐Diff's learning ability for peptides, we counted the frequency of occurrence of amino acids in the peptide sequences generated by CPL‐Diff and compared them with real peptides. As shown in **Figure** **4**, where the left column is the amino acid occurrence frequency distribution of the three real peptides, and the right column is the amino acid occurrence frequency distribution of the three peptides generated by our CPL‐Diff. Overall, the three peptides generated by CPL‐Diff are close to the real peptides in terms of frequency distribution. And it can be noticed that all three peptide sequences generated by CPL‐Diff have a lower tendency for methionine (M). Because M is easily oxidized and M is uncharged, too much M may reduce the activities of AMPs, AFPs, and AVPs. In contrast, the propensity is high for both lysine (K) and leucine (L). Because K is positively charged and L is hydrophobic, an increase in K and L may favor an increase in the activity of AMPs, AFPs, and AVPs. And this phenomenon was also observed for all three real peptides. This suggests that our CPL‐Diff may be able to learn the common phenomenon that the number of occurrences of a particular amino acid has an effect on the activity of most peptides. And we can also notice some special phenomena:

1. For histidine (H), the propensity of both CPL‐Diff and real polypeptides is low, and CPL‐Diff occurs less frequently than real polypeptides. This may be due to the fact that histidine (H) is hydrophilic, and the presence of too much histidine (H) may cause the peptide to preferentially come into contact with water molecules.
2. For tyrosine (Y), both AMPs and AFPs generated by CPL‐Diff appear at a low frequency, whereas they will appear at a higher frequency in generated AVPs than in generated AMPs and AFPs. This is consistent with what happens with real peptides. This may be due to the fact that tyrosine (Y) is uncharged, whereas AMPs and AFPs achieve bacterial or fungal killing mainly by penetrating or disrupting cell membranes, in which case they need to interact with the charge on the cell membrane. In contrast, uncharged amino acids like tyrosine (Y) may not be as important for AMPs and AFPs.
3. For aspartic (D), glutamic (E), and glutamine (Q), the same occurs as for tyrosine (Y) and cysteine (C). This may be due to the fact that aspartic (D), glutamic (E), and glutamine (Q) are negatively charged in the pH environment of the body, and that excess aspartic (D), glutamic (E) and glutamine (Q) may diminish the effectiveness of AMPs and AFPs in penetrating or disrupting cell membranes. In contrast, appropriate increases in aspartate (D), glutamate (E) and glutamine (Q) may help AVP interact with positively charged regions of the virus.

Overall, our CPL‐Diff can learn the patterns of amino acid composition in various types of peptides, and potentially also some physicochemical properties, which can then be used to make a tendency selection based on the physicochemical properties.

##### Alpha Helix and Beta Sheet Propensity

**Figure 5:**
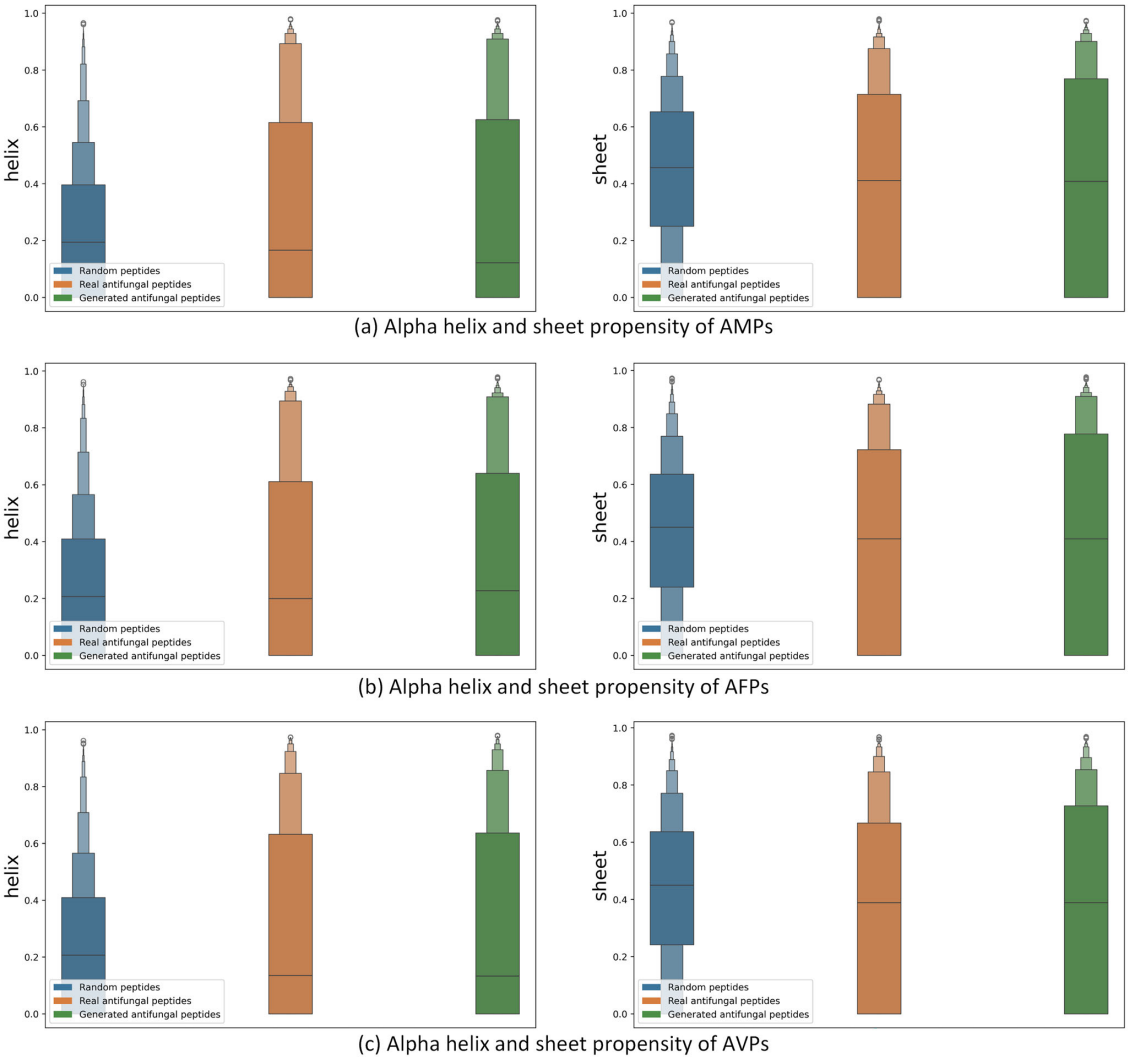
Distribution of Alpha helix and Beta sheet propensity. a), b), and c) Alpha helix and Beta sheet propensity distribution maps for AMPs, AFPs, and AVPs, respectively. The left column is the Alpha helix propensity distribution map and the right column is the Beta sheet propensity distribution map.

We used the Chou‐Fasman method^[^ ^69^ ^]^ to predict the secondary structures of the real polypeptide sequences and the sequences generated by CPL‐Diff, and calculated the Alpha helices and sheet propensities to evaluate the accuracy and reliability of our model. As shown in **Figure** **5**. CPL‐Diff‐generated peptides are very close to those of real peptides in terms of Alpha helix and Beta sheet propensities, which indicates that our CPL‐Diff captures the key structural features of real peptide sequences better. And since the similarity scores of the peptides generated by CPL‐Diff are low (part 4.4.1), this may indicate that CPL‐Diff is capable of discovering new, functionally similar peptide sequences with potential applications.

##### Latent Space Visualization

In order to evaluate the ability of CPL‐Diff to learn latent space embeddings, we used t‐SNE^[^ ^70^ ^]^ and UMAP^[^ ^71^ ^]^ approaches to reduce the dimensionality of generated ESM2 latent space embeddings and ESM2 latent space embeddings of real peptides for visualization. Where the real peptide latent space embedding uses the entire peptide sequence data we collected and samples 1000 samples as the generated peptide latent space embedding respectively.

**Figure 6:**
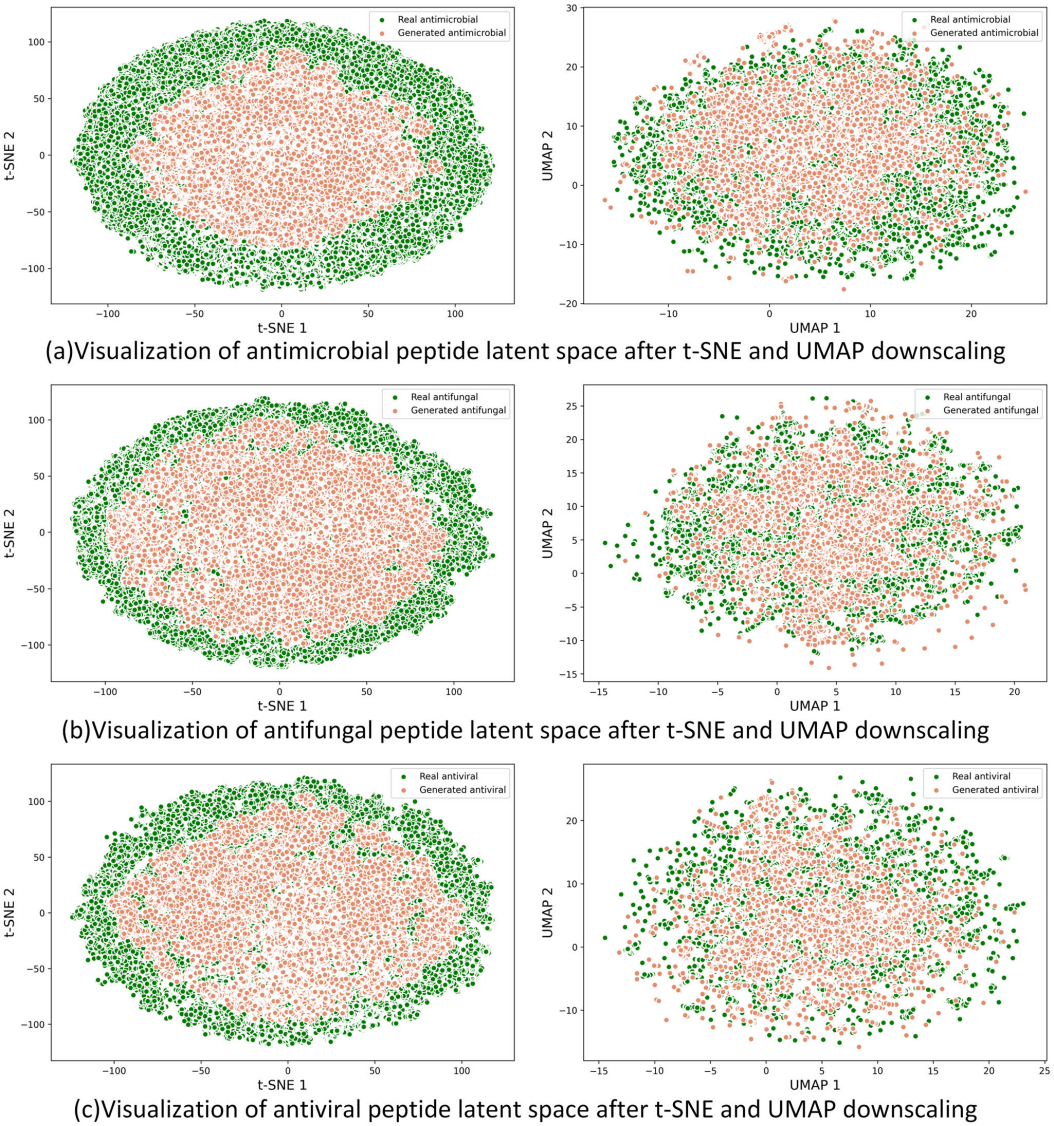
Visualization of the t‐SNE and UMAP dimensionality reduction results for the ESM2 latent space embeddings of real peptides and those generated by CPL‐Diff. Panels a), b), and c) show the dimensionality reduction visualization effects for AMPs, AFPs, and AVPs latent space embeddings, respectively. The left column represents t‐SNE dimensionality reduction, while the right column represents UMAP dimensionality reduction. Green dots represent the real peptide ESM2 latent space embeddings after dimensionality reduction, and red dots represent the CPL‐Diff‐generated ESM2 latent space embeddings after dimensionality reduction.

**Figure** **6** illustrates the results of t‐SNE and UMAP downscaling, showing the peptide embeddings generated by real peptides and CPL‐Diff in the 2D ESM latent space. It can be seen that the peptide latent space embeddings generated by CPL‐Diff exhibit obvious aggregation and obvious overlap phenomenon with the real peptide embeddings, and have significant similarity in distribution compared with the real peptides. This indicates that our CPL‐Diff can capture the intrinsic features of real peptides well.

#### 3.5.3 Ablation Study

To investigate the necessity of each module in CPL‐Diff, we compared CPL‐Diff with its variants: (1) CPL‐Diff(w/o pLM)replaces ESM2 8 M with a common embedding layer; (2) CPL‐Diff(w/o condition)removes the condition guidance module; (3) CPL‐Diff(w/o mask control)removes the mask control generation length module; (4) CPL‐Diff (w/o condition & mask control) removes both the conditional guidance module and the mask‐controlled generation length module. For the CPL‐Diff models without the conditional guidance module, we trained three separate models using the three types of peptide datasets. All models were sampled to generate 1000 peptide sequences for comparison. If the model contains the conditional module, the guidance strengths are consistent with that described in part 3.5.1.

The results are presented in **Table** **5**. When the ESM2 8 M is replaced with a standard embedding layer (w/o pLM), if trained using the CPL‐Diff architecture, the model struggles to learn the features of peptide sequences, resulting in performance nearly equivalent to completely random sequence generation. This indicates that the combination of pre‐trained pLM with diffusion models significantly enhances the quality of generated peptide sequences.

**Table 5:** Ablation study on CPL‐Diff. Each cell represents the metrics for AMPs, AFPs, and AVPs, respectively, separated by slashes. “↑” indicates that the higher the metric, the better. “↓”indicates that the lower the metric, the better. (*n* = 1000).

Methods	Perplexity↓	pLDDT↑	Instability↓	Similarity↓	Activity↑
CPL‐Diff (w/o pLM)	22.3206/ 21.6211/ 22.9786	57.6021/ 57.9181/ 58.0037	45.1446/ 43.4712/ 48.2478	36.4182/ 36.0015/ 35.1470	0.1750/ 0.1540/ 0.1610
CPL‐Diff (w/o condition & mask control)	12.1410/ 11.0439/ 13.9343	66.6493/ 65.9577/ 68.5213	41.4120/ 41.3547/ 48.9931	31.2002/ 27.0132/ 26.8445	0.8850/ 0.8630/ 0.8120
CPL‐Diff (w/o condition)	11.8766/ 10.7276/ 13.5306	66.9977/ 66.4553/ 69.5768	40.9808/ 45.6917/ 46.1866	31.6384/ 27.1412/ 26.5893	0.8880/ 0.8660/ 0.8260
CPL‐Diff (w/o mask control)	11.8254/ 10.7033/ 13.5398	67.1042/ 69.0103/ 70.9471	40.5988/ 42.3264/ 46.9630	31.8037/ 32.0284/ 26.7171	0.9600/ 0.8620/ 0.7240
CPL‐Diff	10.7180/ 10.4099/ 12.8759	68.4545/ 70.1558/ 71.4834	38.3809/ 36.2060/ 44.6779	32.4894/ 32.9795/ 27.2248	0.9720/ 0.8780/ 0.7580

Removing either the mask‐controlled generation length module or the conditional guidance module from CPL‐Diff, or both, leads to a noticeable increase in perplexity and instability compared to the complete CPL‐Diff model, along with a significant decrease in pLDDT scores. Although there is only a slight reduction in similarity, this comes at the cost of increased uncertainty and instability. Furthermore, the frequency distribution of amino acids in the generated sequences shows a significant difference compared to the real frequency distribution (Figures S3–S6, Supporting Information).

Furthermore, it was observed that when utilizing the conditional guidance module, the proportion of active AMPs and AFPs within the generated peptide sequences is higher than when training CPL‐Diff solely on AMPs or AFPs. Conversely, the proportion of active AVPs within the generated sequences is lower than when training CPL‐Diff specifically on AVPs. This may be attributed to the fact that AMPs, AFPs, and AVPs are all infection‐fighting peptides sharing certain biological characteristics, allowing the conditional guidance module to capture commonalities among different types of peptides. However, due to the relatively smaller dataset size for AVPs compared to the other two types, when these three types of peptides are combined, CPL‐Diff learns fewer specific characteristics of AVPs compared to those of AMPs and AFPs.

#### 3.5.4 CPL‐Diff can Control the Length of the Generated Peptide Sequence

Since our CPL‐Diff additionally introduces an attention mask during training (Part 2.2.1), we can actually achieve the goal of having our CPL‐Diff output a specified sequence length by using an attention mask. In this part, we will focus on the performance of CPL‐Diff in controlling the length of the generated sequences.

##### CPL‐Diff Controls the Length of the Generated Sequence Through an Attention Mask

**Figure 7:**
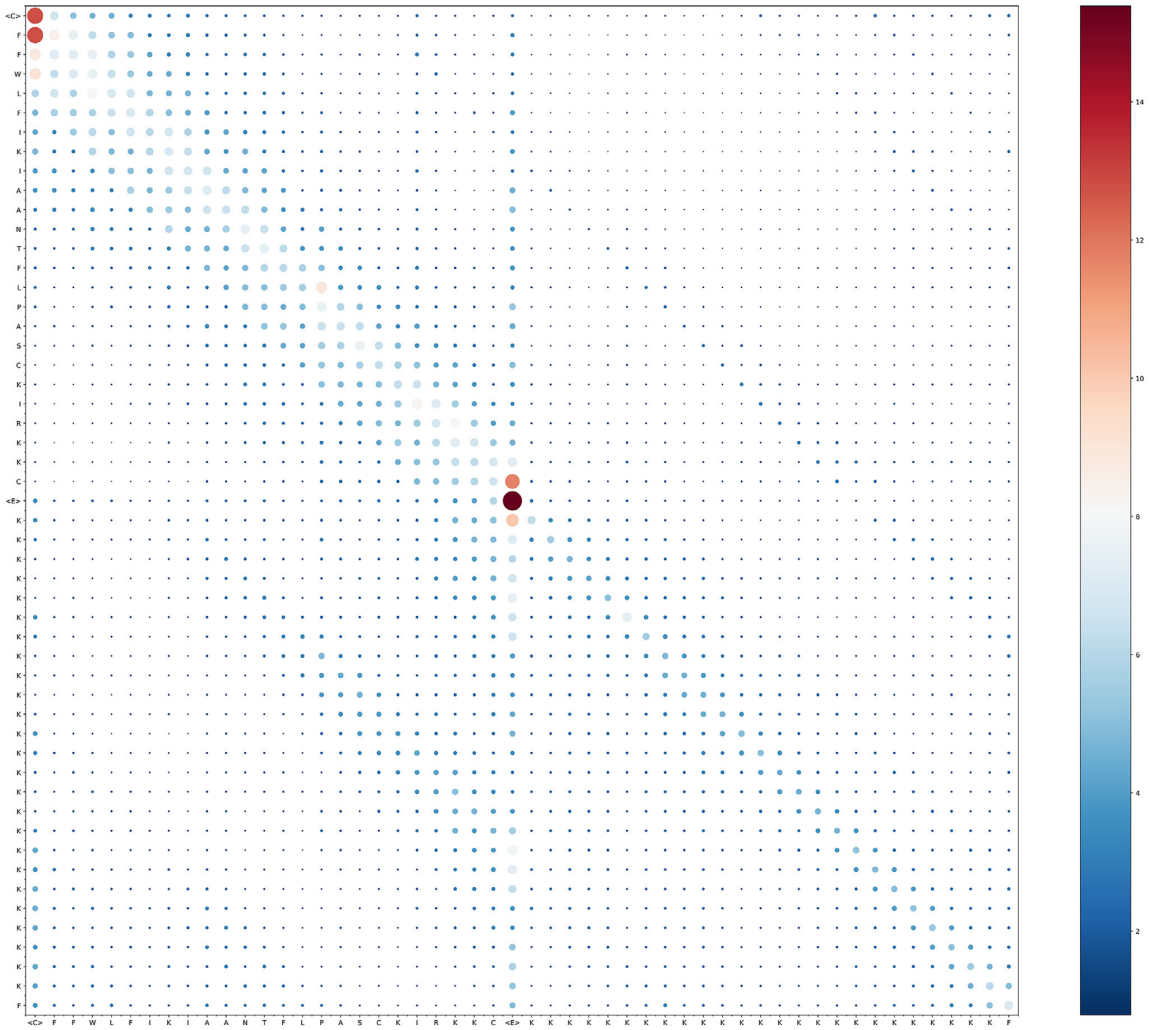
Visualization of the sum of all the attention weight matrices extracted from CPL‐Diff. The bluer the color and the smaller the area of the circle indicate a lower attention score. Redder colors and larger circles indicate higher attention scores. <C> denotes the start marker CLS and <E> denotes the end marker EOS.

To explore how CPL‐Diff carries out the control of the length of the generated sequence, we let CPL‐Diff generate an AMP sequence of length 24. The attention weight matrix of CPL‐Diff was extracted while processing the last time step. All the obtained attention weight matrices were then summed up for global interpretation. This is shown in **Figure** **7**. We can see that the start marker CLS and the end marker EOS have higher attention scores than all the others, indicating that our CPL‐Diff can correctly understand the need to control the length of the generated sequence and add these two markers at the right locations. We can also find some attention scores near the diagonal, indicating that our CPL‐Diff can understand the importance of the relative position of amino acids for peptides. And we can also see that the attention score between leucine (L_14_, i.e., the 14th amino acid in the sequence is L) and proline (P_15_) is higher relative to the rest of the amino acid combinations that are not on the diagonal. The leucine residues, which have the ability to form hydrogen bonds, and the proline residues, which have hydrophobic properties, may make an important contribution to the conformational stability of the AMP, especially when there are synergistic hydrophobic interactions to enhance its linkage integrity. Taken together, these results suggest that CPL‐Diff not only controls the length of the generated sequence, but also has the ability to generate peptides with stable conformations within a limited length range.

##### CPL‐Diff Generates New Peptide Sequences that Strictly Correspond to the Number and Length of Real Peptide Datasets

In part 3.5.1, since the baseline model does not have the ability to control the length of the generated sequences. Therefore, for a fairer comparison, we chose to let CPL‐Diff simulate randomly determining the length of the generated sequences. And in that part, in order to further evaluate the ability of CPL‐Diff in modeling complex biomolecular structures, we let CPL‐Diff generate new polypeptide sequences with lengths and quantities identical to the three real polypeptide sequences, respectively. And the generated peptide sequences are compared with the real peptide sequences. The guide strengths used to generate the three polypeptide sequences are consistent with those mentioned in part 3.5.1.

**Figure 8:**
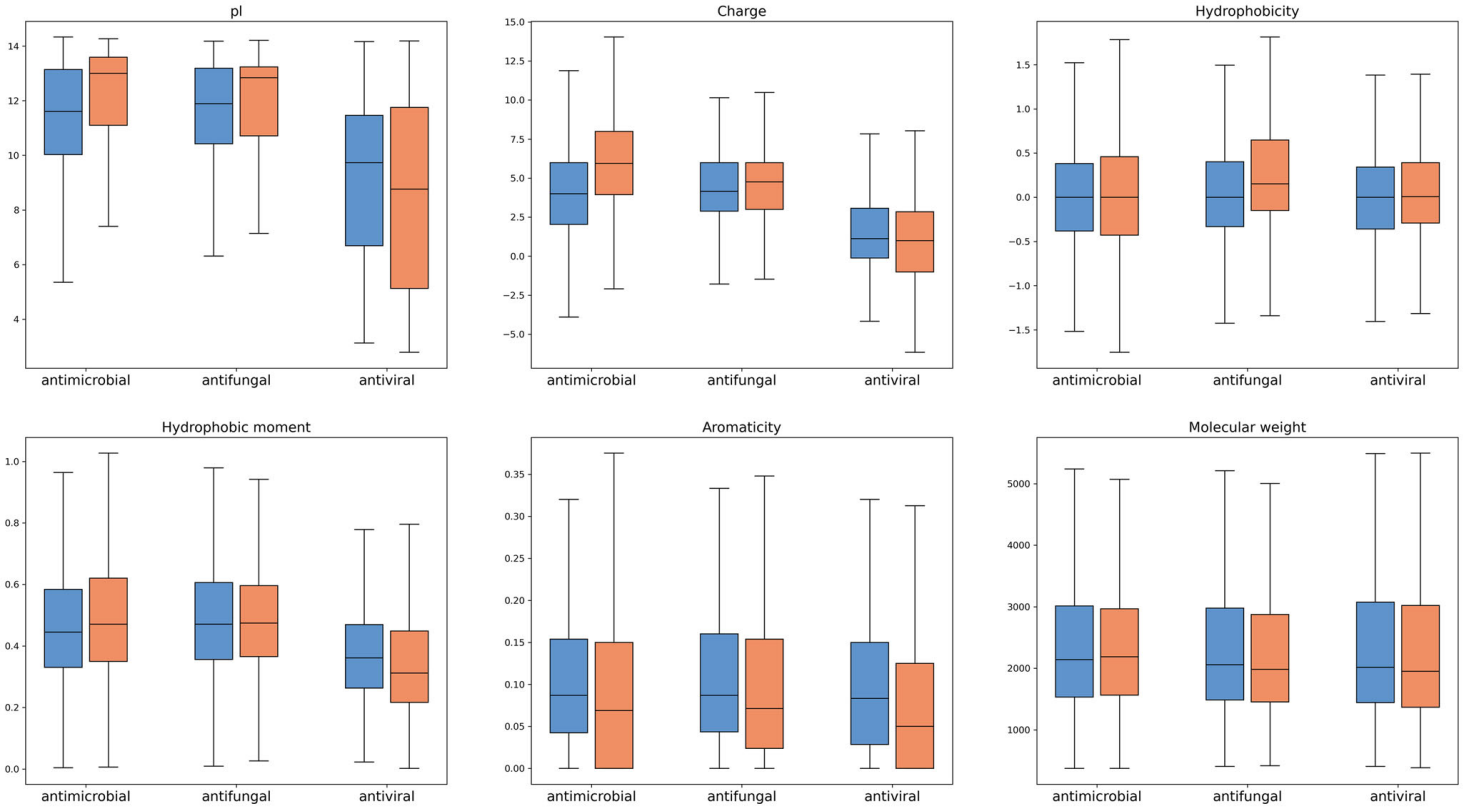
The model generates the distribution of physicochemical properties (including isoelectric point (pI), charge, hydrophobicity, hydrophobic moment, aromaticity, and molecular weight) of peptides whose quantity and length are identical to those of the real polypeptide and the real polypeptide. Each box‐and‐line plot is divided into three sections, AMP, AFP, and AVP. Real peptides are shown on the left side of each section, and CPL‐Diff‐generated peptides are shown on the right side.

The results are shown in **Table** **6** and **Figure** **8**. With strict agreement in length and number, the AMPs and AFPs generated by CPL‐Diff had lower instability scores and lower similarity scores. As for the physicochemical properties, the AMPs and AFPs generated by CPL‐Diff are not only better than the real AMPs and AFPs in terms of isoelectric point, charge, hydrophobicity, hydrophobic moment, and aromaticity, but also have a smaller molecular weight than the real AMPs and AFPs. As for AVPs, although the AVPs generated by CPL‐Diff had higher instability scores than the real AVPs, the lowest similarity scores were achieved. In terms of physicochemical properties, although the isoelectric point and charge are not as high as those of real AVPs, the lower quartile of hydrophobicity is higher than that of real AVPs, as well as the maximum point of hydrophobicity moment, and the upper quartile and maximum point of aromaticity are higher than that of real AVPs, whereas in terms of molecular weights of AVPs, although the upper quartile and the maximum point of the molecular weights of AVPs generated by CPL‐Diff are higher than that of real AVPs. quartile points were slightly higher than the real AVPs, their median and lower quartile points were lower than the real AVPs. The above results further demonstrate that CPL‐Diff not only has the ability to simulate the structure of complex biomolecules, but also has the ability to generate peptides that are more active, easier to synthesize and more novel.

**Table 6:** The model generates a comparison of results with quantities and lengths that are identical to those of real peptides. Where the left side of the slash indicates the results for real peptides and the right side of the slash indicates the results for CPL‐Diff generated peptides. “Activate” indicates the proportion of the generated sequence predicted to be active.

Model	Perplexity	Instability	Similarity	Activity
Antimicrobial	15.3703/10.6080	41.6195/40.0444	–/32.3530	–/0.9646
Antifungal	14.6803/10.1577	41.5572/37.6440	–/32.7490	–/0.8841
Antiviral	17.7406/13.2622	46.0613/44.0303	–/27.2057	–/0.7623

##### CPL‐Diff Possesses Strong Generalization Capabilities

Peptides of different lengths will have different physicochemical properties and may have different mechanisms of action, modes of action, and effects. And in practical applications, it may be necessary to generate peptides with specific lengths for specific infection scenarios.^[^ ^58^ ^]^ And theoretically, the distribution of all data is close to the standard Gaussian distribution after a certain degree of diffusion operation. In other words, an initial Gaussian noise would theoretically correspond to peptide sequences of all lengths. To further explore the flexibility and creativity of CPL‐Diff, we choose to let CPL‐Diff generate peptide sequences of different lengths under the same initial noise. And the random noise used in the denoising process for generating sequences of different lengths is all the same.

Taking AMP as an example, we select a part of the results to show (**Table** **7**), and the complete results for the other two peptides and AMP are shown in Tables S4–S6 (Supporting Information). It can be seen that CPL‐Diff, i.e., with the same initial noise, still generates sequences that meet the length requirements. Moreover, as the length of the sequence increases, CPL‐Diff does not directly add amino acids to the end of the sequence to form a longer sequence based on a shorter sequence, but rather adds amino acids to the existing amino acids after some modifications. For example, for a polypeptide sequence of length 5, it can be seen that the AMP score is less than 0.5. However, when the length of the sequence is increased by 1, not only is the amino acid composition changed considerably, but also its AMP score is greater than 0.5. This is probably due to the fact that there are fewer real AMPs for length 5 than for length 6 (Figure S2, Supporting Information), and that a short sequence's exploration space would be smaller than for longer sequences. This would somewhat make CPL‐Diff relatively less likely to generate AMP sequences of length 5. However, overall, this behavior suggests that CPL‐Diff may have some intelligence and learning ability to generate more complex and diverse AMP sequences.

**Table 7:** CPL‐Diff results of generating AMP sequences of different lengths with the same initial noise. SAMP denotes the AMP score obtained by the prediction tool on top of CAMPR4. SAMP < 0.5 indicates that the prediction is non‐AMP. SAMP ≥ 0.5 indicates that the prediction is AMP.

Sequence	Length	S_AMP_	Instability	pI	Charge	Hydrophobic moments	Aromaticity	Molecular weights
MVGRA	5	0.49	−8.98	13.5508	1.99	0.7137	0	531.68
RVRRVR	6	0.6	103.8	13.8633	4.99	0.5682	0	840.04
RVWRVRI	7	0.56	8.5714	13.7969	3.99	0.3972	0.1429	983.22
RVRIVRIRRV	10	0.69	57.79	13.9141	5.99	0.5413	0	1321.67
RVRIVRIRRVR	11	0.71	53.4455	13.9551	6.99	0.5070	0	1477.85
GVGIVKIGRILGRGR	15	0.95	−8.0467	13.8008	4.989	0.7121	0	1549.92
WVGIVKIVRVLGRGRR	16	0.96	6.2313	13.8633	5.989	0.7454	0.0625	1863.31
GVLSALIGAIAGAGHHAHSLIKRK	24	0.96	9.0167	13.5664	4.114	0.3144	0	2376.82
GVLSALIGAIAGAGKHAHSAAKYKH	25	0.97	−3.984	11.0527	4.112	0.3191	0.04	2427.82
GVLSKLIGKIAGAGKKAASSAKKKIS	26	1	−2.5692	13.2656	7.986	0.4888	0	2511.08
GVLSTKIGSIAGAGASAASSILSKISKSCLC	31	0.98	27.8774	10.4277	3.68	0.3645	0	2880.4
SVCSCKISSILGCICPCTSSSVCSISGICVKC	32	0.9	49.2375	7.9097	1.757	0.2501	0	3170.89
SVCSCKICSILGPCCPCTSSSVCSISGICVKYC	33	0.87	39.9273	7.8312	1.601	0.2150	0.0303	3334.09
SVCIAKIPSILGPHHPCHSSIKYCISGHGLKIGSRKVCKR	40	0.93	28.78	10.5862	7.536	0.3609	0.025	4311.2
KVCIAKIPSILGPHHPCNSSIKYCISGKGLKIGSRKVCCRK	41	0.94	8.7366	10.6073	9.298	0.3663	0.0244	4423.44
KVCIAKIGSILGNGHPCNSSIKYCISGAGVKIGGRKGCCRKW	42	0.98	0.9762	10.4775	8.256	0.3677	0.0476	4363.26
KKKIAKIGSIAGVGAGGTGSIVGSIAGAGVGIGGAIGGLIGKGIKCAC	48	0.88	−1.6417	10.9800	6.679	0.3026	0	4194.05
KKKIAKIGSIAGGGAGGAGSIVGSIAGAGVGIGGAIGGLIGKGIKKAKK	49	0.9	−1.8694	13.3125	9.985	0.3260	0	4300.18
MKPIAKIGSIAGAGAGATGSIVGSIAGAGVGIGGAIGGLIGAGIKKADKK	50	0.79	−13.94	11.3740	5.987	0.2827	0	4388.21

We can also find that its instability score does not increase with the length of the sequence, i.e., there is no fixed pattern. For example, the sequence of length 6 has an instability score of 103.8, while the sequence of length 50 has an instability score of ‐13.94. This is because the stability of a polypeptide is influenced by a combination of factors rather than being mainly determined by the sequence length to determine it. As well as due to the complexity of biomolecular systems, CPL‐Diff's results may still have some errors and uncertainties. However, it also shows that CPL‐Diff has taken into account a variety of factors affecting the stability and is able to model the complex relationship between these factors to a certain extent.

#### 3.5.5 Simulated Docking Experiment

To evaluate the ability of CPL‐Diff to capture key structural features of peptides and to further demonstrate the feasibility of CPL‐Diff, we use ESMFold^[^ ^21^ ^]^ to predict the structure of CPL‐Diff generated sequences. Specific targets are then selected for simulated docking experiments. And combined with attention visualization for quantitative analysis. We use the ZDOCK tool^[^ ^72^ ^]^ to perform docking and evaluate the docking scores. The docking results are then visualized using the pymol open source version (v3.0.0).

**Figure 9:**
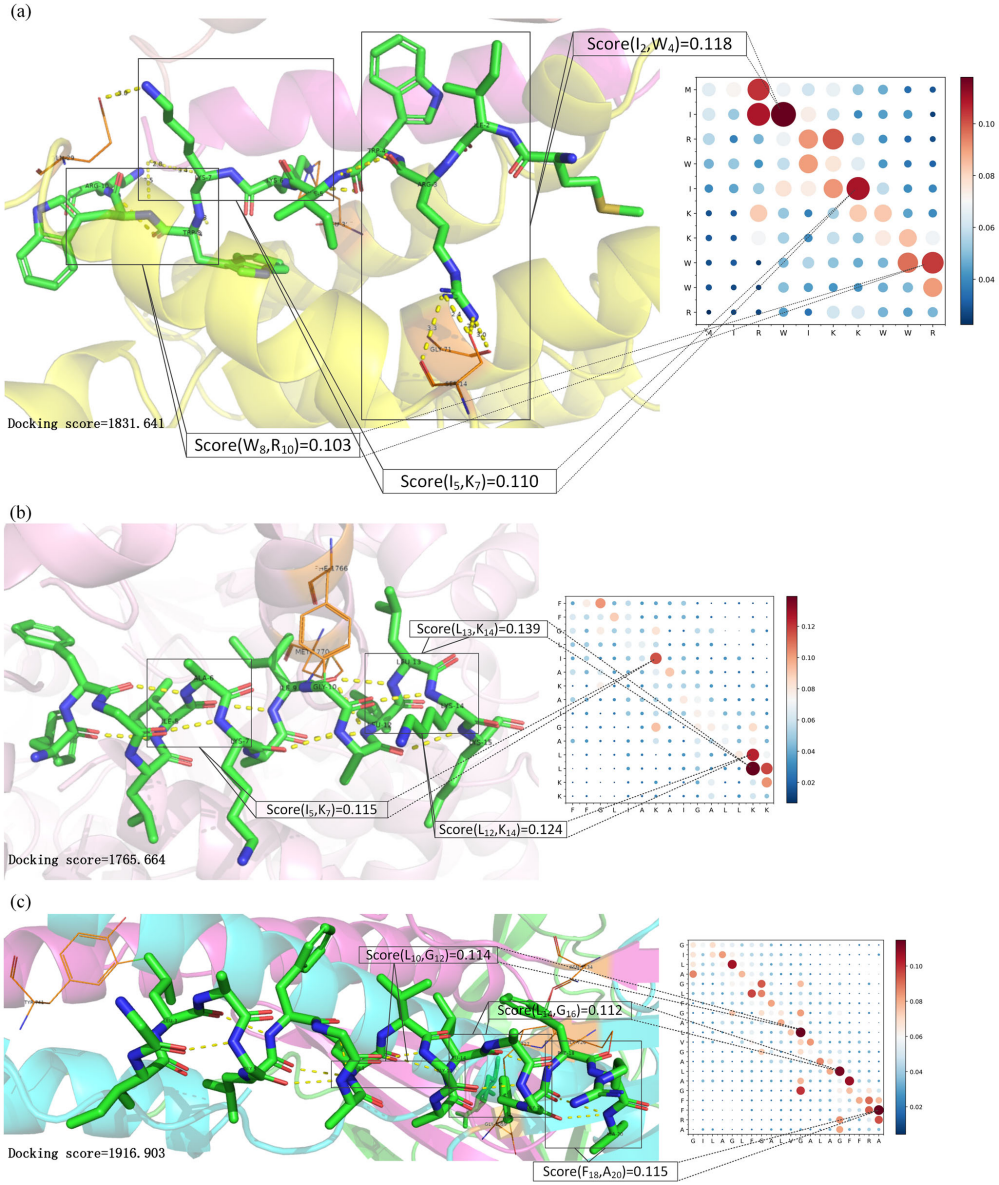
Quantitative analysis of docking of peptide sequences generated by CPL‐Diff. The structures corresponding to the generated peptide sequences were predicted using ESMFold and docking was simulated using ZDock. a) Docking quantification of AMP sequences generated by CPL‐Diff. b) Docking quantification of AFP sequences generated by CPL‐Diff. c) Docking quantification of CPL‐Diff generated AVP sequences. (a), (b) and (c) The left half shows the docking results visualized using pymol, where the bottom right corner is the docking score given by ZDock. The right half shows the extracted attention weight matrix visualization. Here, only the sequence part is visualized, ignoring the start marker, the end marker, and the part after the end marker. Thick green lines indicate residues of the peptide and thin orange lines indicate residues of the target protein.

For the AMP, we chose lipopolysaccharide^[^ ^73^ ^]^ from the bacterial outer membrane as the target protein for molecular docking. We let CPL‐Diff generate an AMP sequence with sequence length 10 and perform mock docking experiments. The docking results are shown in **Figure** **9**a. We can see that the combination of isoleucine (I) at the second position and tryptophan (W) at the fourth position (I_2_,W_4_) has the highest attention score. Both isoleucine (I) and tryptophan (W) are hydrophobic and a strong hydrophobic interaction may be formed between them. This interaction helps in the localisation and penetration of the AMP in the bacterial membrane, thus helping the arginine (R) at the third position to contact the target protein. And for (I_5_,K_7_), we can find that the lysine (K) at the seventh position makes contact with the hydrogen bond of the target protein. This may be because the combination of lysine (K), although hydrophilic, with isoleucine (I), which is hydrophobic, may help the AMP to localise and penetrate the cell membrane. Also we can see that the lysine (K) at the sixth position makes a non‐polar contact with the target protein. For (W_8_,R_10_) it can be seen that a hydrogen bond is formed between these two amino acids, which helps to increase the stability of the peptide structure. The docking results without heatmaps are provided in Figure S7 (Supporting Information).

For AFP, we chose 1,3‐β glucan^[^ ^74^ ^]^ on the fungal outer membrane as the target protein for molecular docking. We let CPL‐Diff generate the AFP sequence with sequence length 15 and perform mock docking experiments. The docking results are shown in Figure 9b. It can be seen that (L_13_,K_14_) and (L_12_,K_14_), which have the highest attention, are a combination of leucine (L), which is hydrophobic, and lysine (K), which is hydrophilic and positively charged. This combination may render the AFP amphiphilic, allowing the antifungal peptide to be stable in an aqueous environment and to interact with fungal cell membranes. Hydrogen bonds are formed between (I_5_,K_7_), which could help the AFP maintain a stable structure. Isoleucine (I) at the ninth position and glycine (G) at the tenth position both make non‐polar contacts with the target protein. The docking results without heatmaps are provided in Figure S8 (Supporting Information).

For the AVP, we chose the SARS‐CoV‐2 spike protein^[^ ^75^ ^]^ as the target protein for molecular docking. We let CPL‐Diff generate an AVP sequence with a sequence length of 20 and perform mock docking experiments. The docking results are shown in Figure 9c. It can be seen that the combination of phenylalanine (F) at the eighteenth position and alanine (A) at the twentieth position (F_18_,A_20_) has the highest attention score. Both phenylalanine (F) and alanine (A) are hydrophobic and can form hydrophobic regions, which can promote the binding of AVP to target proteins. And the small side chain of alanine (A) makes it easy to interact with other amino acids, thus promoting the formation of aromatic interactions. It can also be seen from Figure 9c that the phenylalanine (F) at the eighteenth position has a nonpolar contact with the target protein. And both the phenylalanine (F) at the eighteenth position and the alanine (A) at the twentieth position formed hydrogen bonds with other amino acids. And for (L_10_,G_12_) and (L_14_,G_16_), both leucine (L) and glycine (G) are hydrophobic, which can form hydrophobic regions and thus promote the binding of AVP to target proteins. In contrast, both leucine (L) and glycine (G) are non‐polar amino acids, which may allow them to form stable interactions in certain conformations. For example, van der Waals forces between the aromatic ring of leucine (L) and the small side chain of glycine (G) may increase the stability of the peptide. And whereas the flexibility of glycine (G) contributes to peptide folding and stabilization. The docking results without heatmaps are provided in Figure S9 (Supporting Information).

The above results show that the peptide sequences generated by our CPL‐Diff exhibit good binding ability and bioactivity in docking experiments, which suggests that CPL‐Diff has a high predictive ability to generate peptides of high quality. And the quantitative analysis of the simulation experiments helps to understand which amino acid pairs have important interactions in the generation process, which is expected to reveal the key factors of peptide sequence design and provide important theoretical guidance for the design of peptides.

## 4 Conclusion and Future Work

This paper presents the DDPM‐based CPL‐Diff model for generating multiple peptide sequences. The model uses an attention mask to control the length of the generated sequences and incorporates a protein language model to and use classifierless bootstrapping to generate peptide sequences with three different functions (antibacterial, antifungal, and antiviral). In contrast to previous work, CPL‐Diff is able to control the length of the generated sequences using only an attention mask, eliminating the need to sample from a fitted polynomial distribution as initial noise, thus broadening the space for exploration. The contributions of this work include the following:(1) The application of the masking mechanism to the diffusion model used to generate peptide sequences, which can control the length of the generated peptide sequences without relying on any distribution. (2) Using multiple types of peptide sequences to train the model so that the model captures the commonality of different peptides. And most of the previous methods use a single type of peptide for training. CPL‐Diff can use conditional information to guide the generation of therapeutic peptides with different effects, thus reducing the training cost. (3) Interpretability analysis of our model not only provides a better understanding of how CPL‐Diff controls the length of the generated sequences, but also helps us understand how CPL‐Diff generates specific peptide sequences. It is expected to reveal the key factors of peptide sequence design and provide important theoretical guidance for the design of peptides.

However, due to certain limitations of Denoising Diffusion Probabilistic Models (DDPMs), which require thousands of iterations to obtain final results, the generation process demands substantial computational time. To address this, we propose to employ more efficient sampling strategies in the reverse process, such as Denoising Diffusion Implicit Models (DDIMs). Additionally, the inherent class imbalance in our dataset's polypeptide types may lead to uneven learning across different categories in CPL‐Diff. To mitigate this issue, we intend to implement sample balancing techniques during the training phase, including adjusting the mixing weights between conditional and unconditional generation to enhance generation propensity for rare target categories. Furthermore, in subsequent research endeavors, we intend to extend this methodology to encompass the generation of alternative non‐infectious peptides (e.g., anticancer peptides) while enhancing its capability to simultaneously generate protein sequences with specified lengths and their corresponding structural configurations through attention masking mechanisms.

## Conflict of Interest

The authors declare no conflict of interest.

## Supporting information



Supporting Information

## Data Availability

The data that support the findings of this study are available from the corresponding author upon reasonable request.
