# Supplementary material for: CPL‐Diff: A Diffusion Model for De Novo Design of Functional Peptide Sequences with Fixed Length
Source: Adv Sci (Weinh). 2025 Apr 15;12(20):2412926. doi: 10.1002/advs.202412926 (PMC12120732; doi:10.1002/advs.202412926)
Supplement: Supplementary file 1 — Supporting Information [file ADVS-12-2412926-s001.docx]

Supporting Information

CPL-Diff: A Diffusion Model for De Novo Design of Functional Peptide Sequences with Fixed Length

*Zhenjie Luo^1^, Aoyun Geng^1^, Feifei Cui^1^, Leyi Wei^2,3^, Quan Zou^4,5^, Zilong Zhang^1,*^*

1. Hyperparametric sensitivity analysis

In Part 3.4, we present the hyperparameters employed for model training. Here, we conduct a systematic sensitivity analysis focusing on six critical hyperparameters: noise scheduling strategy, maximum timestep (T), number of attention layers, quantity of attention heads, learning rate (lr), and learning rate schedule. All results were computationally derived from 1,000 randomly generated peptide sequence samples of varying lengths, produced by their respective models.

1.1 Noise schedule analysis

We first investigated the performance variations of CPL-Diff under distinct noise schedules, conducting comparative analyses of linear, cosine, and square-root (sqrt) configurations. The experimental configuration maintained fixed parameters including a learning rate of 10^-3^ with cosine decay scheduling, maximum timestep T=1000, 6 attention layers, and 20 attention heads per layer. Results are summarized in Table S1.

It can be observed that the proportion of active sequences generated by CPL-Diff under varying numbers of attention layers exhibited no significant disparity. Although CPL-Diff under the linear and cosine noise schedules demonstrated lower perplexity and diversity compared to the sqrt schedule, their diversity (Entropy) was notably inferior to that of CPL-Diff under the square root schedule. Most importantly, CPL-Diff under the sqrt schedule exhibited lower instability and reduced error margins. Therefore, the sqrt noise schedule was selected as the optimal configuration.

**Table S1.** Performance evaluation of peptide sequences generated by CPL-Diff under different noise scheduler. Each cell represents the metrics (mean ± standard deviation) for antimicrobial peptides, antifungal peptides, and antiviral peptides, respectively, separated by slashes.

| Hyperparametric | Perplexity | Entropy | Instability | Similarity | Activate |
| --- | --- | --- | --- | --- | --- |
| linear | 7.9806±4.0580/  7.7548±3.6649/  8.0928±4.7826 | 2.0625±0.8018/  2.1864±0.7847/  1.9788±0.8139 | 45.8036±72.6740/  42.5070±79.9025/  56.0580±70.1512 | 30.0716±9.8289/  31.1257±10.2052/  23.3362±8.6428 | 0.9360/  0.8780/  0.7530 |
| cosine | 9.5133±4.5108/  8.8236±4.2945/  10.5871±5.3169 | 2.3072±0.8040/  2.3794±0.8216/  2.3274±0.8261 | 39.3947±56.7904/  36.3927±71.9628/  45.2035±46.4297 | 31.2982±10.0244/  31.8094±10.3841/  25.2139±9.1052 | 0.9460/  0.8780/  0.7010 |
| sqrt | 10.9642±4.7746/  10.3740±4.5303/  13.5435±4.8354 | 2.4321±0.7889/  2.5050±0.7790/  2.6713±0.6186 | 41.0957±56.3238/  37.7398±72.3011/  44.1536±42.1190 | 32.0823±10.0630/  32.6597±10.3408/  27.3053±8.7866 | 0.9520/  0.8640/  0.7350 |

1.2 Attention layer quantity analysis

We next conduct an analysis of the attention layer quantity selection. Comparative evaluations were performed across configurations containing 5, 6, and 7 attention layers. The experimental protocol maintained fixed hyperparameters including a learning rate of 10^-3^ with cosine decay scheduling, maximum timestep T=1000, sqrt noise scheduling, and 20 attention heads per layer. Results are summarized in Table S2 and S3.

**Table S2.** Performance evaluation of peptide sequences generated by CPL-Diff under different attention layer quantity. Each cell represents the metrics (mean ± standard deviation) for antimicrobial peptides, antifungal peptides, and antiviral peptides, respectively, separated by slashes.

| Hyperparametric | Perplexity | Entropy | Instability | Similarity | Activate |
| --- | --- | --- | --- | --- | --- |
| 5 | 10.7298±4.6629/  9.9453±4.3209/  13.2912±4.8941 | 2.3991±0.7719/  2.4678±0.7923/  2.6472±0.6096 | 42.7316±60.3586/  44.3352±81.1311/  45.4288±42.0987 | 31.8546±10.1112/  32.1596±10.2129/  26.9353±8.4533 | 0.9610/  0.8710/  0.7390 |
| 6 | 10.9642±4.7746/  10.3740±4.5303/  13.5435±4.8354 | 2.4321±0.7889/  2.5050±0.7790/  2.6713±0.6186 | 41.0957±56.3238/  37.7398±72.3011/  44.1536±42.1190 | 32.0823±10.0630/  32.6597±10.3408/  27.3053±8.7866 | 0.9520/  0.8640/  0.7350 |
| 7 | 11.7618±4.7168/  10.8872±4.3577/  14.0446±5.0068 | 2.5222±0.7471/  2.5977±0.7417/  2.7143±0.5933 | 38.7831±51.7201/  37.4789±69.3251/  47.3337±47.0808 | 32.3869±10.0527/  32.9819±10.3171/  27.5814±8.6777 | 0.965/  0.865/  0.741 |

It can be observed that the proportion of active sequences generated by CPL-Diff under varying numbers of attention layers exhibited no significant disparity. Furthermore, as the number of layers increased, perplexity and diversity (Entropy) displayed marginal increments, while instability decreased and sequence similarity improved. However, physicochemical evaluations in Table S3 revealed that configurations with 6 attention layers achieved superior performance in isoelectric point, charge, and hydrophobic moment compared to those with 5 or 7 attention layers. Although aromaticity metrics for the 6 attention layers configuration were slightly lower than those for the 5 and 7 attention layers counterparts, their variance was substantially reduced, indicating enhanced stability. This observation may stem from the limited representational capacity of ESM2 8M. While larger ESM2 variants could potentially improve performance with increased attention layers, the associated growth in parameter count would inevitably reduce sampling efficiency. Collectively, to balance generation quality and computational speed, we selected 6 attention layers as the optimal configuration.

**Table S3.** Physicochemical properties evaluation of peptide sequences generated by CPL-Diff under different attention layer quantity. Each cell represents the metrics (mean ± standard deviation) for antimicrobial peptides, antifungal peptides, and antiviral peptides, respectively, separated by slashes.

| Hyperparametric | pI | Charge | Hydrophobic moments | Aromaticity |
| --- | --- | --- | --- | --- |
| 5 | 11.9685±2.0612/  11.9233±1.9118/  7.1237±3.4258 | 5.5508±3.2664/  4.6869±2.8784/  -0.4295±3.5550 | 0.4777±0.2045/  0.5049±0.1995/  0.3187±0.1379 | 0.1378±0.1681/  0.1396±0.1751/  0.0965±0.1187 |
| 6 | 12.0464±2.0279/  12.0440±1.7876/  7.8814±3.4708 | 5.5518±3.4886/  4.9464±2.8487/  0.3167±3.2232 | 0.4858±0.2094/  0.5099±0.1969/  0.3229±0.1510 | 0.1249±0.1535/  0.1226±0.1515/  0.0897±0.1153 |
| 7 | 11.8406±2.0938/  11.8488±1.7785/  7.3438±3.4099 | 5.0865±3.2159/  4.5354±2.4294/  -0.1157±3.3753 | 0.4720±0.1909/  0.4986±0.1816/  0.3174±0.1395 | 0.1252±0.1385/  0.1263±0.1455/  0.0946±0.1050 |

1.3 Attention heads quantity analysis

Next, we analyze the selection of the quantity of attention heads per layers. SThe multi-head attention mechanism employed here partitions input sequences into sub-vectors of identical dimensionality. This design enables each head to learn diverse attention patterns, thereby capturing richer contextual dependencies. Additionally, parameter matrices are shared across all heads, improving computational efficiency. Such implementations are widely adopted in modern Transformer architectures (e.g., ESM2). However, this approach imposes a divisibility constraint: the input dimensionality must be evenly divisible by the number of heads. Given that the latent variable dimensionality of the selected ESM2 8M framework is 320, we evaluated configurations with 10, 16, 20, and 32 attention heads per layer. The experimental protocol maintained fixed hyperparameters including a learning rate of 10^-3^ with cosine decay scheduling, maximum timestep T=1000, sqrt noise scheduling, and 6 attention layers. Results are presented in Tables S4 and S5.

**Table S4.** Performance evaluation of peptide sequences generated by CPL-Diff under different attention heads quantity. Each cell represents the metrics (mean ± standard deviation) for antimicrobial peptides, antifungal peptides, and antiviral peptides, respectively, separated by slashes.

| Hyperparametric | Perplexity | Entropy | Instability | Similarity | Activate |
| --- | --- | --- | --- | --- | --- |
| 10 | 10.8403±4.6263/  9.9000±4.2882/  12.9976±5.0706 | 2.4253±0.7780/  2.4798±0.7690/  2.6194±0.6087 | 44.2889±61.2477/  39.5778±74.1552/  50.1701±46.1765 | 32.1033±9.9294/  32.5037±10.1707/  27.2404±8.8195 | 0.9570/  0.8700/  0.7770 |
| 16 | 11.1490±4.7850/  10.4669±4.3748/  14.2687±5.0930 | 2.4781±0.7904/  2.5525±0.7901/  2.7340±0.6030 | 41.5416±55.3641/  38.6400±71.2380/  45.3903±43.0555 | 32.3167±10.0656/  32.8115±10.2476/  27.6437±8.8366 | 0.9660/  0.8840/  0.7420 |
| 20 | 10.9642±4.7746/  10.3740±4.5303/  13.5435±4.8354 | 2.4321±0.7889/  2.5050±0.7790/  2.6713±0.6186 | 41.0957±56.3238/  37.7398±72.3011/  44.1536±42.1190 | 32.0823±10.0630/  32.6597±10.3408/  27.3053±8.7866 | 0.9520/  0.8640/  0.7350 |
| 32 | 11.5473±4.8443/  10.7795±4.5453/  14.4762±5.1313 | 2.4936±0.7779/  2.5599±0.7736/  2.7564±0.5581 | 44.5376±58.6854/  40.2745±75.6270/  47.7445±44.5892 | 32.4116±10.1421/  32.7684±10.2688/  27.8792±8.6939 | 0.9550/  0.8630/  0.7200 |

**Table S5.** Physicochemical properties evaluation of peptide sequences generated by CPL-Diff under different attention heads quantity. Each cell represents the metrics (mean ± standard deviation) for antimicrobial peptides, antifungal peptides, and antiviral peptides, respectively, separated by slashes.

| Hyperparametric | pI | Charge | Hydrophobic moments | Aromaticity |
| --- | --- | --- | --- | --- |
| 10 | 11.9755±2.0741/  11.9369±1.8334/  7.7617±3.2556 | 5.5076±3.3250/  4.8244±2.8801/  0.3093±3.2954 | 0.4928±0.2090/  0.5117±0.1979/  0.3410±0.1658 | 0.1265±0.1535/  0.1174±0.1542/  0.0809±0.1014 |
| 16 | 11.9440±2.0266/  11.9205±1.8641/  7.2479±3.3687 | 5.4313±3.2544/  4.7321±3.0135/  -0.1120±3.3616 | 0.5035±0.2055/  0.5298±0.1962/  0.3324±0.1507 | 0.1298±0.1496/  0.1207±0.1476/  0.0975±0.1136 |
| 20 | 12.0464±2.0279/  12.0440±1.7876/  7.8814±3.4708 | 5.5518±3.4886/  4.9464±2.8487/  0.3167±3.2232 | 0.4858±0.2094/  0.5099±0.1969/  0.3229±0.1510 | 0.1249±0.1535/  0.1226±0.1515/  0.0897±0.1153 |
| 32 | 12.0076±2.0344/  11.8557±1.8842/  7.2561±3.2681 | 5.3800±3.1360/  4.6146±2.5927/  -0.0710±3.0343 | 0.4973±0.2050/  0.5157±0.1999/  0.3390±0.1538 | 0.1297±0.1466/  0.1168±0.1464/  0.0951±0.1134 |

It can be observed that the proportion of active sequences generated by CPL-Diff under different numbers of attention heads does not exhibit significant disparities. Furthermore, the results indicate that when employing 20 attention heads per layer, although the model achieves suboptimal perplexity and slightly reduced diversity (Entropy) compared to configurations with 16 and 32 attention heads, it demonstrates the lowest instability index and comparatively lower sequence similarity. In terms of physicochemical properties, the 20-head configuration manifests optimal comprehensive performance. This phenomenon may be attributed to the model's over-reliance on noise within the training data rather than generalized features when the number of attention heads exceeds a critical threshold, ultimately leading to compromised generalization capacity. Based on systematic evaluation of these quantitative metrics, we ultimately selected the configuration with 20 attention heads per layer as the optimal architecture.

1.4 Noise timestep analysis

We next conduct an analysis of the maximum timestep selection. In diffusion model, determining the maximum noise timestep constitutes a critical hyperparameter for fine-grained control of generation dynamics. Our investigation evaluates three configurations with maximum timestep T values set to 500, 1000, and 2000. The experimental protocol maintained fixed hyperparameters including a learning rate of 10^-3^ with cosine decay scheduling, sqrt noise scheduling, 6 attention layers, and 20 attention heads per layer. Results are summarized in Table S6 and S7.

**Table S6.** Performance evaluation of peptide sequences generated by CPL-Diff under different maximum timestep. Each cell represents the metrics (mean ± standard deviation) for antimicrobial peptides, antifungal peptides, and antiviral peptides, respectively, separated by slashes.

| Hyperparametric | Perplexity | Entropy | Instability | Similarity | Activate |
| --- | --- | --- | --- | --- | --- |
| 500 | 10.9136±4.4874/  10.0503±4.2331/  13.3355±4.7902 | 2.4553±0.7468/  2.4936±0.7661/  2.6660±0.6129 | 43.6702±59.3992/  40.8395±73.9951/  45.3193±44.0836 | 32.3799±10.2279/  32.6563±10.4854/  27.3650±8.6850 | 0.9510/  0.8790/  0.7010 |
| 1000 | 10.9642±4.7746/  10.3740±4.5303/  13.5435±4.8354 | 2.4321±0.7889/  2.5050±0.7790/  2.6713±0.6186 | 41.0957±56.3238/  37.7398±72.3011/  44.1536±42.1190 | 32.0823±10.0630/  32.6597±10.3408/  27.3053±8.7866 | 0.9520/  0.8640/  0.7050 |
| 2000 | 10.7606±4.5126/  10.4722±4.2276/  12.9764±5.2668 | 2.4212±0.7534/  2.5210±0.7522/  2.6128±0.6169 | 40.0102±55.6500/  35.9740±68.0967/  46.1481±44.3330 | 32.0701±10.0374/  32.5657±10.2748/  27.1519±8.8253 | 0.9680/  0.8660/  0.7100 |

Our experimental analysis reveals a progressive enhancement in active sequence ratio and reduction in perplexity across CPL-Diff-generated polypeptides as the maximum timestep (T) increases, accompanied by marginally diminished sequence diversity (Entropy). Notably, sequence similarity demonstrates a gradual decline with increasing timestep. However, antiviral peptides generated under T=2000 exhibit elevated instability indices compared to configurations with T=500 and T=1000, potentially attributable to inherent imbalances in peptide type distribution within the training dataset. Regarding physicochemical characteristics, antimicrobial and antiviral sequences generated at T=2000 manifest superior isoelectric points and charge values compared to other timestep configurations, while antifungal peptide performance remains comparable across all timesteps. All configurations demonstrate equivalent hydrophobic moment and aromaticity metrics. Crucially, the T=2000 configuration achieves lower standard deviations across multiple evaluation metrics, indicative of enhanced generation stability. Based on these comprehensive evaluations, we ultimately selected T=2000 as the optimal maximum timestep configuration.

**Table S7.** Physicochemical properties evaluation of peptide sequences generated by CPL-Diff under different maximum timestep. Each cell represents the metrics (mean ± standard deviation) for antimicrobial peptides, antifungal peptides, and antiviral peptides, respectively, separated by slashes.

| Hyperparametric | pI | Charge | Hydrophobic moments | Aromaticity |
| --- | --- | --- | --- | --- |
| 500 | 11.9066±2.0886/  11.9064±1.8610/  7.8055±3.4013 | 5.5158±3.3900/  4.8278±2.6736/  0.1711±3.1850/ | 0.4825±0.2007/  0.5015±0.1938/  0.3279±0.1501 | 0.1300±0.1514/  0.1290±0.1623/  0.1000±0.1170 |
| 1000 | 12.0464±2.0279/  12.0440±1.7876/  7.8814±3.4708 | 5.5518±3.4886/  4.9464±2.8487/  0.3167±3.2232 | 0.4858±0.2094/  0.5099±0.1969/  0.3229±0.1510 | 0.1249±0.1535/  0.1226±0.1515/  0.0897±0.1153 |
| 2000 | 12.1276±1.9545/  11.9798±1.7383/  8.1232±3.3764/ | 5.7208±3.3252/  4.8107±2.8345/  0.6960±3.1447/ | 0.4838±0.2050/  0.4887±0.1857/  0.3285±0.1563/ | 0.1142±0.1438/  0.1249±0.1609/  0.0760±0.0992/ |

1.5 Training hyperparameter analysis

The training hyperparameters encompass a series of configurations including learning rate, batch size, number of optimization steps, and learning rate decay schedule. Given the extensive combinatorial possibilities inherent in these parameters, we employed the Bayesian optimization method provided by Weights & Biases (Wandb) to perform 100 automated tuning iterations. Through this systematic approach, we ultimately identified the optimal configuration: a learning rate of 9.84×10^-4^, a batch size of 64, and 200,000 optimization steps. For the learning rate annealing schedule, we adopted a warm-up cosine decay strategy, with the learning rate decaying to 10^-5^ over the course of 200,000 optimization steps.


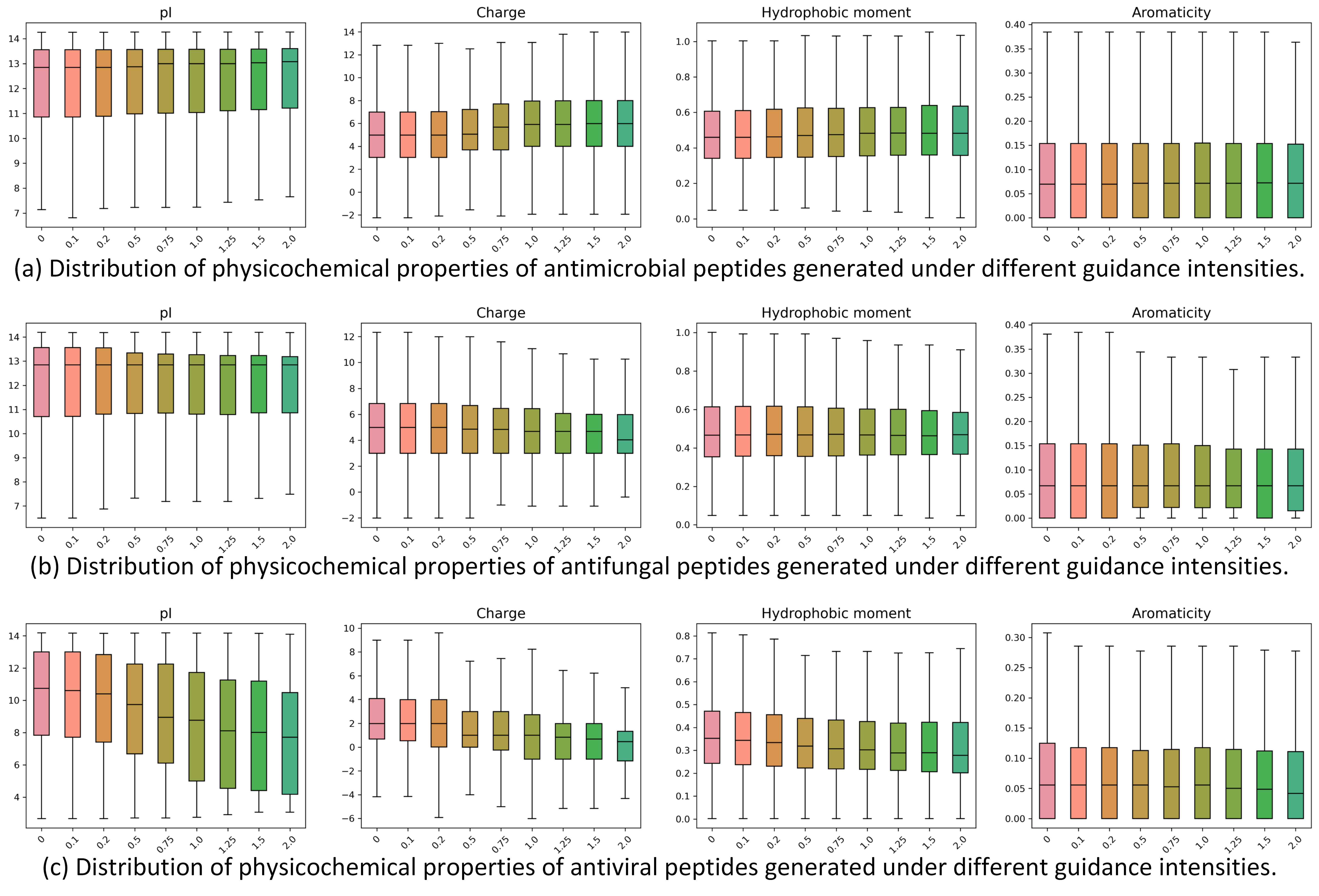


**Figure S1.** Distribution of physicochemical properties (including isoelectric point (pI), charge, hydrophobic moment and aromaticity) of peptide sequences generated by CPL-Diff under different guidance strength. (a) Distribution of physicochemical properties of antimicrobial peptide sequences generated by CPL-Diff under different guidance strength. (b) Distribution of physicochemical properties of antifungal peptide sequences generated by CPL-Diff under different guidance strength. (c) Distribution of physicochemical properties of antiviral peptide sequences generated by CPL-Diff under different guidance strength.


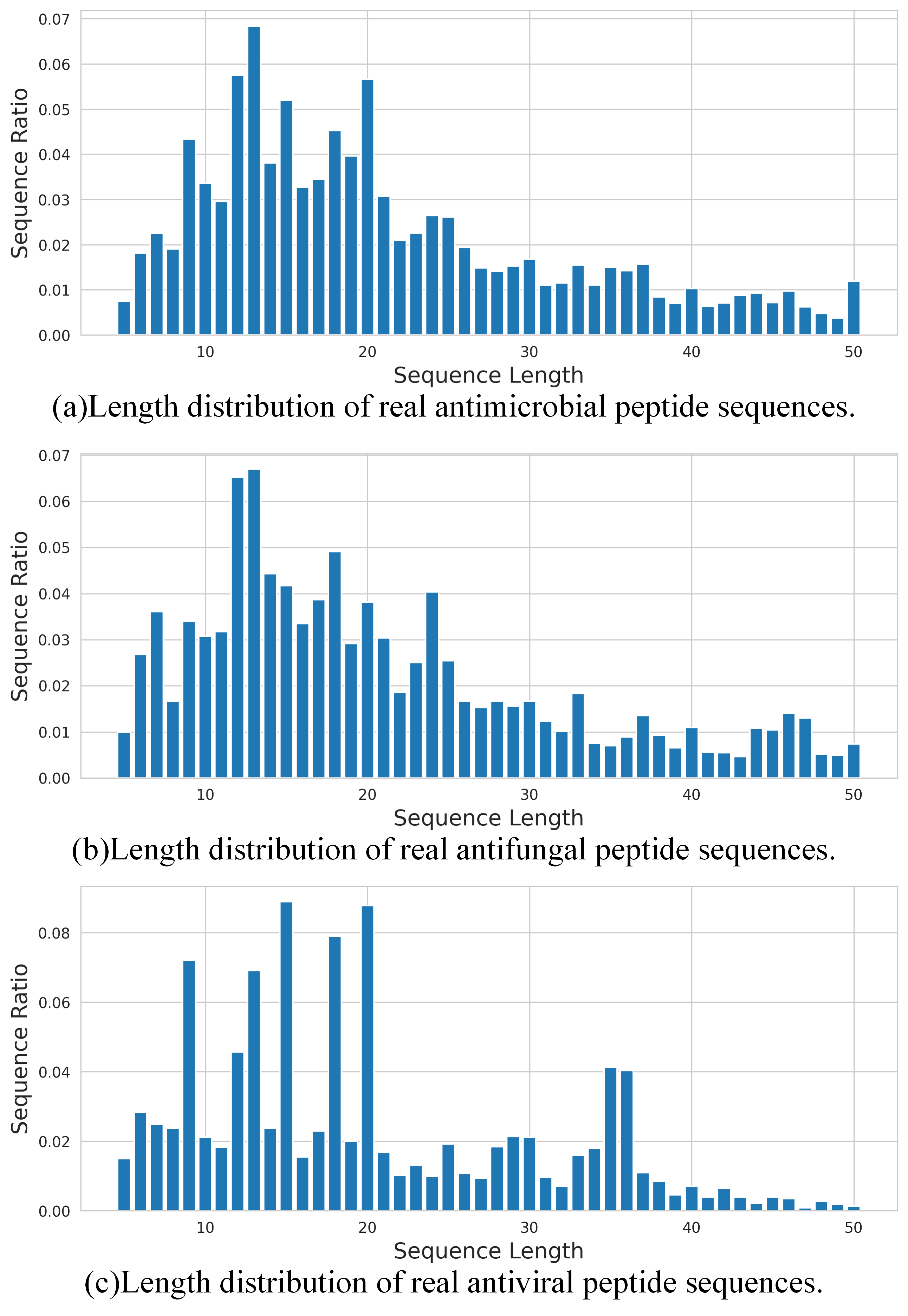


**Figure S2.** Length distribution of real polypeptide sequences. (a) Length distribution of real AMPs sequences. The horizontal coordinate is the length of real AMPs sequences, and the vertical coordinate is the total number of sequences of a certain length as a percentage of the total number of real AMPs. (b) Length distribution of real AFPs sequences. The horizontal coordinate is the length of real AFPs sequences, and the vertical coordinate is the total number of sequences of a certain length as a percentage of the total number of real AFPs. (c) Length distribution of real AVPs sequences. The horizontal coordinate is the length of real AVPs sequences, and the vertical coordinate is the total number of sequences of a certain length as a percentage of the total number of real AVPs.


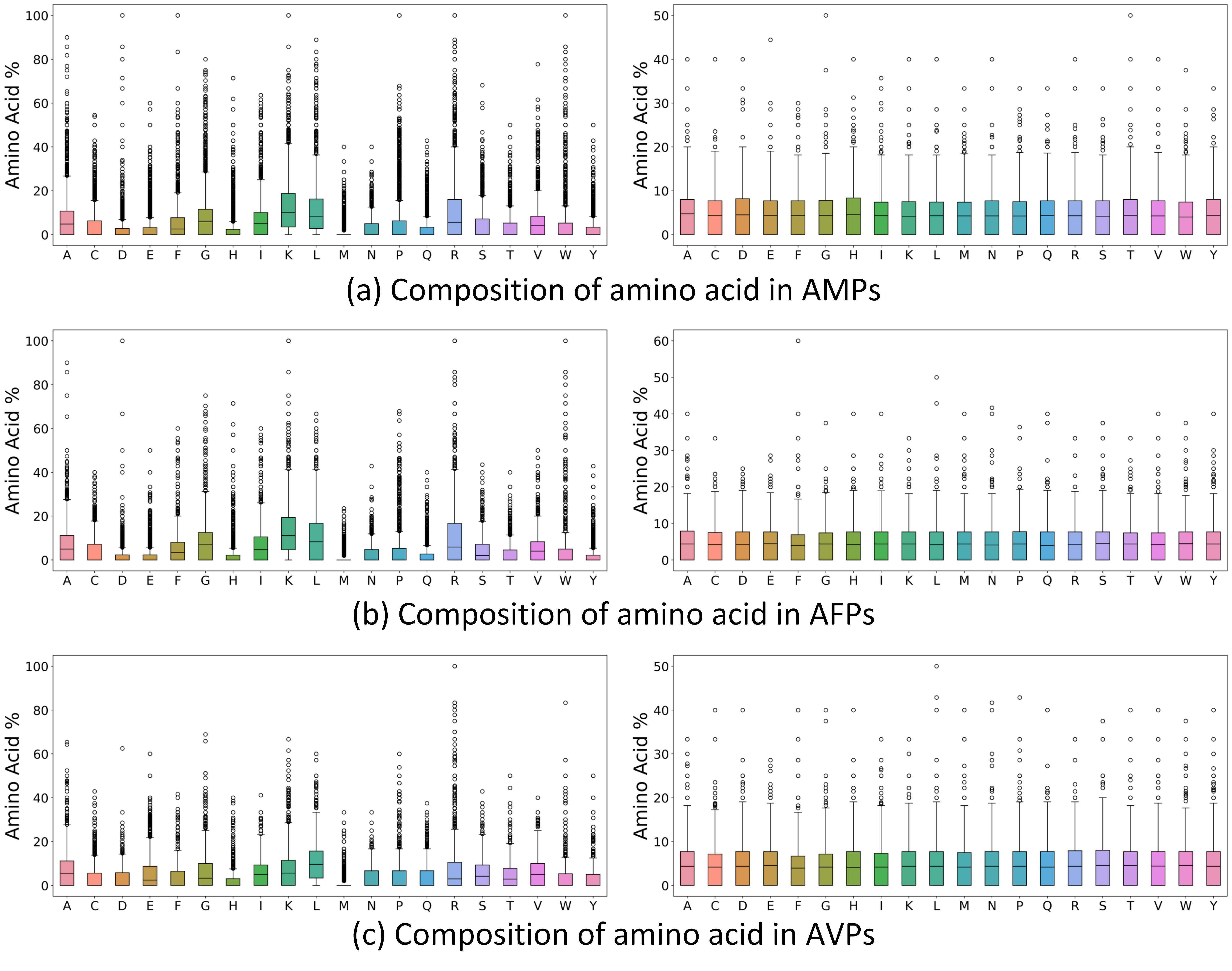


**Figure S3.** Distribution of amino acid occurrence frequencies. a) Amino acid occurrence frequencies of AMPs. Frequency of amino acid occurrence for real AMPs on the left and amino acid occurrence for CPL-Diff (w/o pLM)-generated AMPs on the right. b) Frequency of amino acid occurrence for AFPs. Frequency of amino acid occurrence for real AFPs on the left and amino acid occurrence for CPL-Diff (w/o pLM)-generated AFPs on the right. c) Frequency of amino acid occurrence for AVPs. Frequency of amino acid occurrence of real AVPs on the left and amino acid occurrence of CPL-Diff (w/o pLM)-generated AVPs on the right.


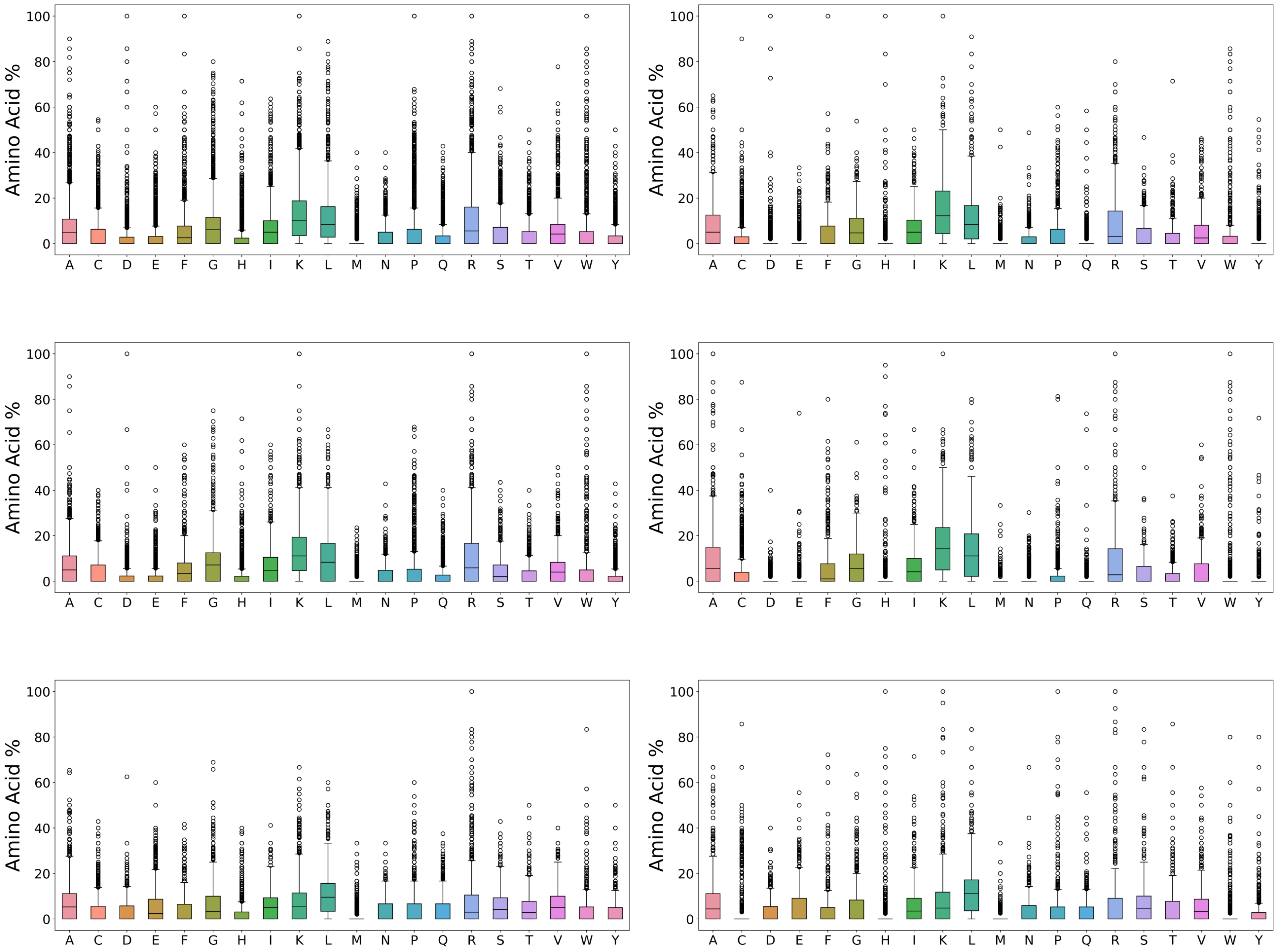


**Figure S4.** Distribution of amino acid occurrence frequencies. a) Amino acid occurrence frequencies of AMPs. Frequency of amino acid occurrence for real AMPs on the left and amino acid occurrence for CPL-Diff (w/o condition & mask control)-generated AMPs on the right. b) Frequency of amino acid occurrence for AFPs. Frequency of amino acid occurrence for real AFPs on the left and amino acid occurrence for CPL-Diff (w/o condition & mask control)-generated AFPs on the right. c) Frequency of amino acid occurrence for AVPs. Frequency of amino acid occurrence of real AVPs on the left and amino acid occurrence of CPL-Diff (w/o condition & mask control)-generated AVPs on the right.


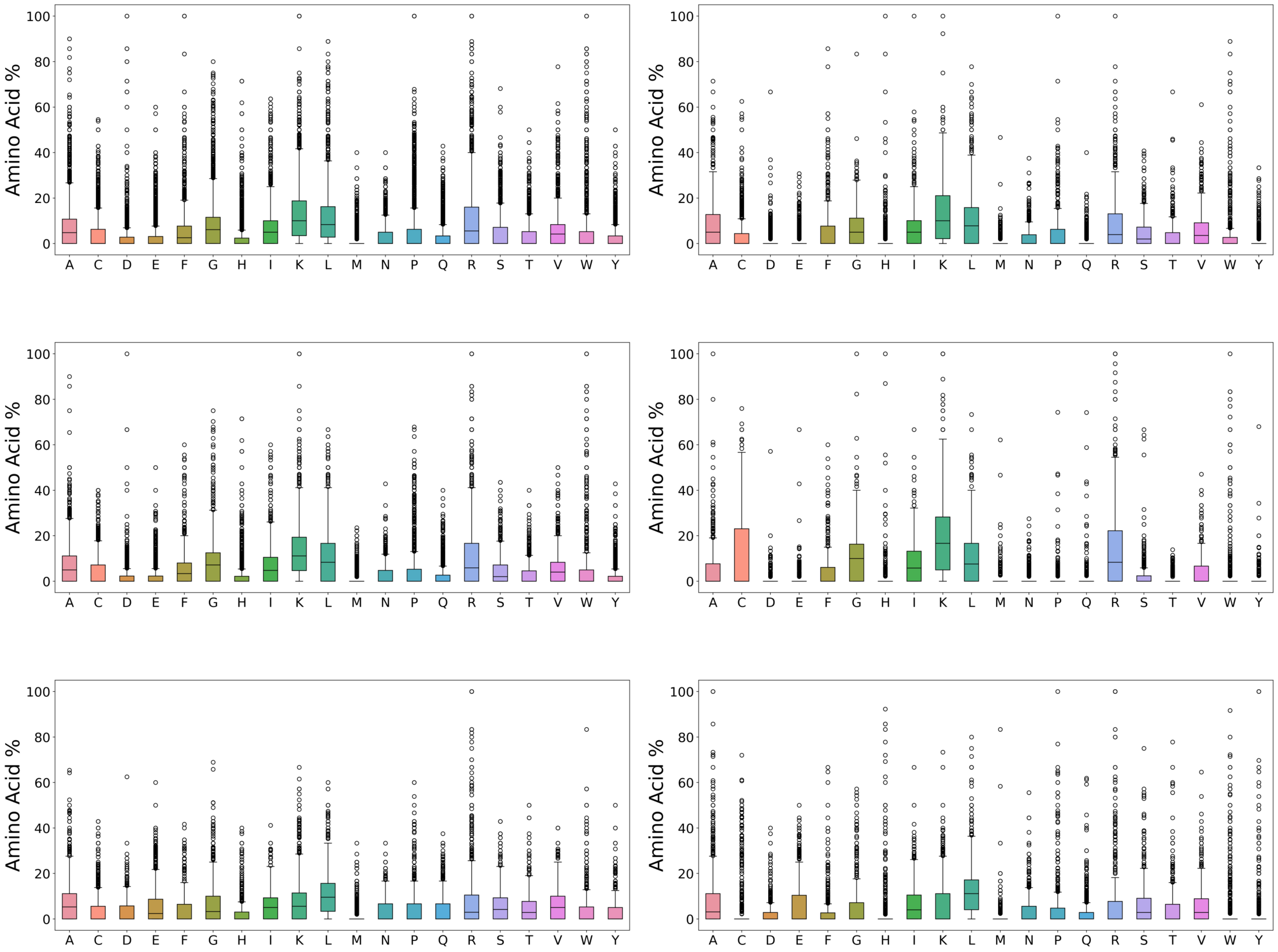


**Figure S5.** Distribution of amino acid occurrence frequencies. a) Amino acid occurrence frequencies of AMPs. Frequency of amino acid occurrence for real AMPs on the left and amino acid occurrence for CPL-Diff (w/o condition)-generated AMPs on the right. b) Frequency of amino acid occurrence for AFPs. Frequency of amino acid occurrence for real AFPs on the left and amino acid occurrence for CPL-Diff (w/o condition)-generated AFPs on the right. c) Frequency of amino acid occurrence for AVPs. Frequency of amino acid occurrence of real AVPs on the left and amino acid occurrence of CPL-Diff (w/o condition)-generated AVPs on the right.


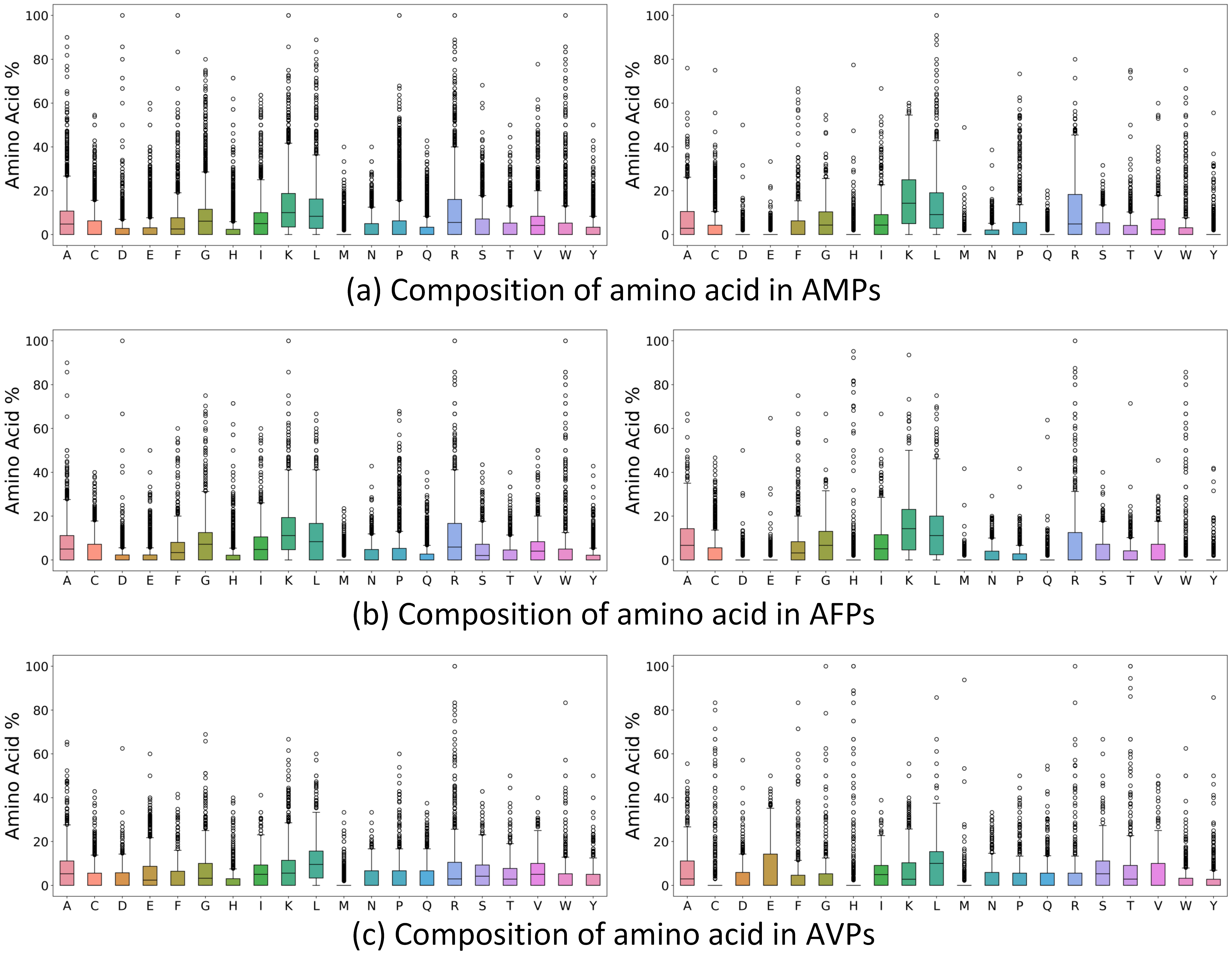


**Figure S6.** Distribution of amino acid occurrence frequencies. a) Amino acid occurrence frequencies of AMPs. Frequency of amino acid occurrence for real AMPs on the left and amino acid occurrence for CPL-Diff (w/o mask control)-generated AMPs on the right. b) Frequency of amino acid occurrence for AFPs. Frequency of amino acid occurrence for real AFPs on the left and amino acid occurrence for CPL-Diff (w/o mask control)-generated AFPs on the right. c) Frequency of amino acid occurrence for AVPs. Frequency of amino acid occurrence of real AVPs on the left and amino acid occurrence of CPL-Diff (w/o mask control)-generated AVPs on the right.


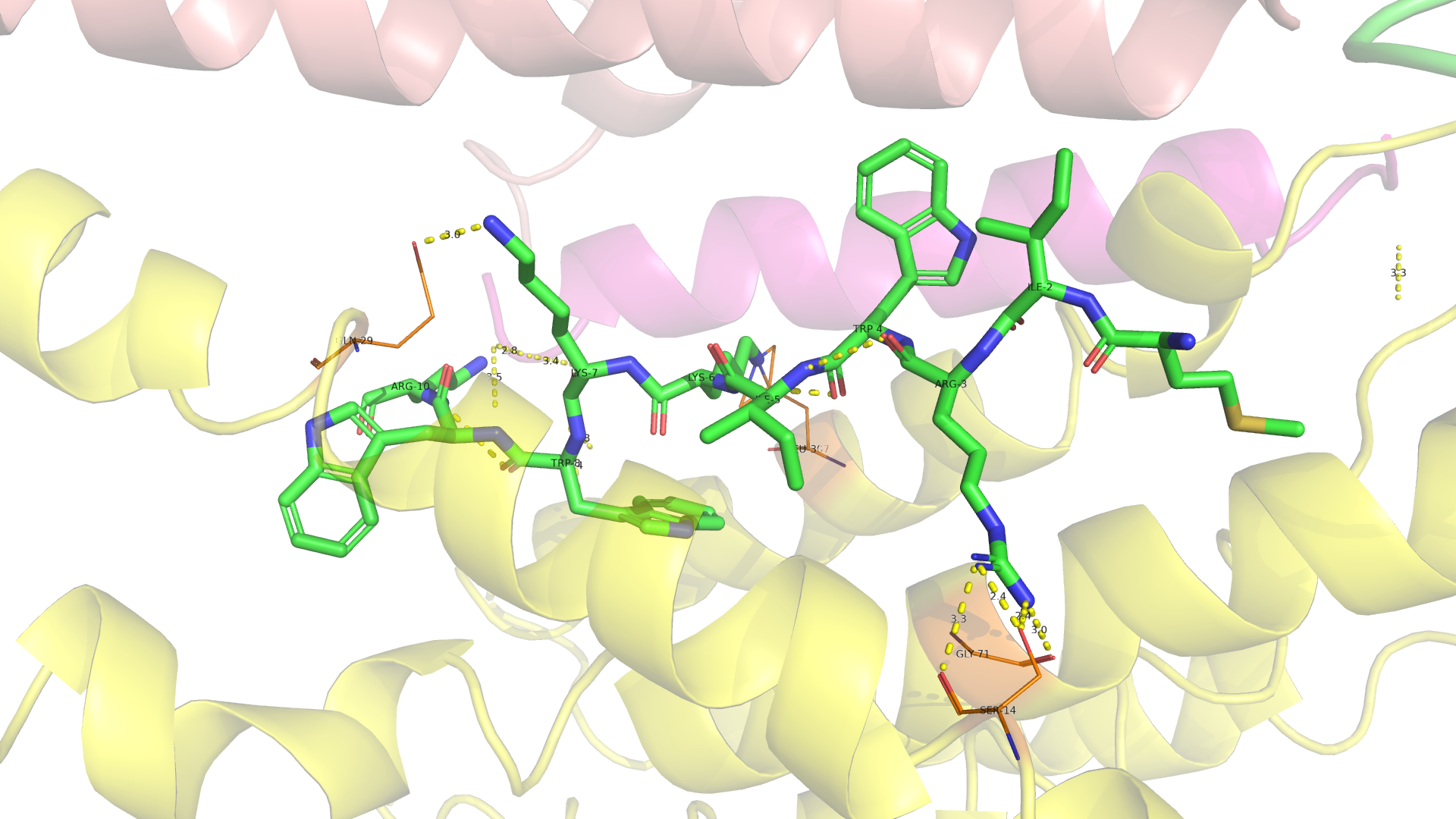


**Figure S7.** Simulated Docking Results of CPL-Diff-Generated Antimicrobial Peptides with Lipopolysaccharides on the Bacterial Outer Membrane.

**
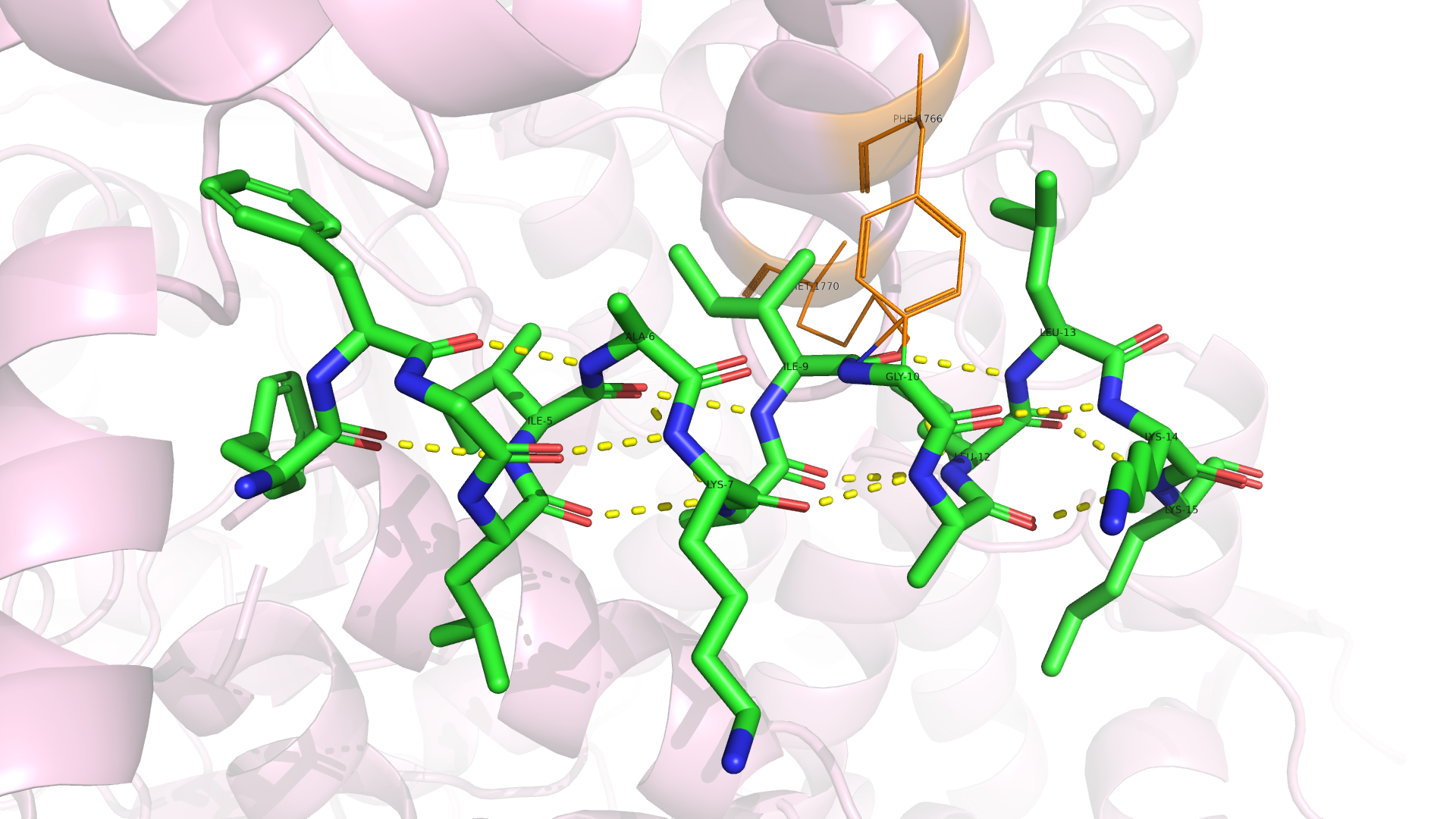
**

**Figure S8.** Simulated Docking Results of CPL-Diff-Generated Antifungal Peptides with 1,3-β-Glucans on the Fungal Outer Membrane.

**
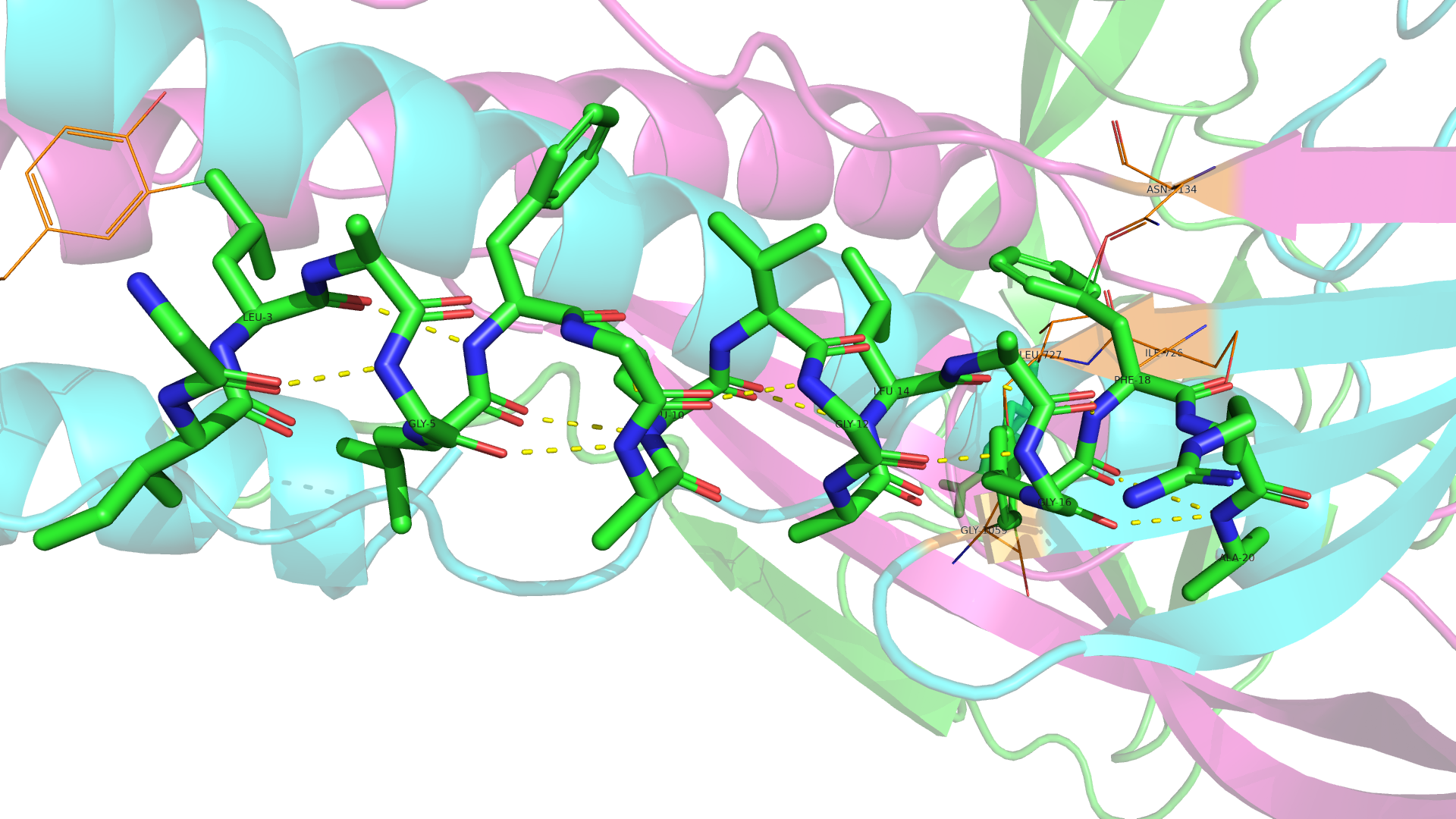
**

**Figure S9.** Simulated Docking Results of CPL-Diff-Generated Antiviral Peptides with the SARS-CoV-2 Spike Protein.

**Table S8.** Performance evaluation of AMP sequences generation by CPL-Diff under different guidance strengths.

| Guidance strength | Perplexity | Entropy | Instability | Similarity | Activity |
| --- | --- | --- | --- | --- | --- |
| $\lambda=0.0$ | 11.3472 | 2.5328 | 39.0950 | 32.3815 | 0.9500 |
| $\lambda=0.1$ | 11.3099 | 2.5275 | 39.0423 | 32.3888 | 0.9530 |
| $\lambda=0.2$ | 11.2833 | 2.5202 | 38.7839 | 32.3997 | 0.9550 |
| $\lambda=0.5$ | 11.1724 | 2.5078 | 38.2297 | 32.4892 | 0.9600 |
| $\lambda=0.75$ | 11.0231 | 2.4910 | 38.2336 | 32.5113 | 0.9650 |
| $\lambda=1.0$ | 10.9019 | 2.4715 | 38.2802 | 32.5174 | 0.9690 |
| $\lambda=1.25$ | 10.7956 | 2.4572 | 38.3099 | 32.5114 | 0.9720 |
| $\lambda=1.5$ | 10.7181 | 2.4391 | 38.3809 | 32.4894 | 0.9730 |
| $\lambda=2.0$ | 10.5874 | 2.4147 | 38.5059 | 32.4760 | 0.9730 |

**Table S9.** Performance evaluation of AFP sequences generation by CPL-Diff under different guidance strengths.

| Guidance strength | Perplexity | Entropy | Instability | Similarity | Activity |
| --- | --- | --- | --- | --- | --- |
| $\lambda=0.0$ | 11.0756 | 2.5700 | 39.6583 | 32.9515 | 0.8290 |
| $\lambda=0.1$ | 11.0071 | 2.5668 | 38.9894 | 32.9771 | 0.8330 |
| $\lambda=0.2$ | 10.9790 | 2.5663 | 38.3817 | 33.0382 | 0.8370 |
| $\lambda=0.5$ | 10.8985 | 2.5724 | 37.5656 | 33.1043 | 0.8550 |
| $\lambda=0.75$ | 10.8229 | 2.5735 | 36.5903 | 33.1601 | 0.8670 |
| $\lambda=1.0$ | 10.6862 | 2.5689 | 35.4769 | 33.1530 | 0.8760 |
| $\lambda=1.25$ | 10.5806 | 2.5663 | 35.3745 | 33.1095 | 0.8760 |
| $\lambda=1.5$ | 10.4099 | 2.5538 | 36.2060 | 32.9795 | 0.8780 |
| $\lambda=2.0$ | 10.1740 | 2.5411 | 34.8724 | 32.8589 | 0.8940 |

**Table S10.** Performance evaluation of AVP sequences generation by CPL-Diff under different guidance strengths.

| Guidance strength | Perplexity | Entropy | Instability | Similarity | Activity |
| --- | --- | --- | --- | --- | --- |
| $\lambda=0.0$ | 13.3183 | 2.6636 | 43.5692 | 27.6584 | 0.7720 |
| $\lambda=0.1$ | 13.3067 | 2.6620 | 43.5997 | 27.5997 | 0.7630 |
| $\lambda=0.2$ | 13.3328 | 2.6663 | 43.5142 | 27.5559 | 0.7580 |
| $\lambda=0.5$ | 13.1822 | 2.6574 | 44.6364 | 27.4336 | 0.7350 |
| $\lambda=0.75$ | 12.9896 | 2.6453 | 44.9022 | 27.2925 | 0.7350 |
| $\lambda=1.0$ | 12.8759 | 2.6403 | 44.6779 | 27.2248 | 0.7270 |
| $\lambda=1.25$ | 12.7663 | 2.6199 | 45.3411 | 27.1068 | 0.7240 |
| $\lambda=1.5$ | 12.6754 | 2.6091 | 45.4851 | 27.0734 | 0.7330 |
| $\lambda=2.0$ | 12.4701 | 2.5798 | 45.7259 | 26.9680 | 0.7330 |

**Table S11.** CPL-Diff results of generating AMP sequences of different lengths with the same initial noise. S_AMP_ denotes the AMP score obtained by the prediction tool on top of CAMPR4. S_AMP_ < 0.5 indicates that the prediction is non-AMP. S_AMP_ ≥ 0.5 indicates that the prediction is AMP.

| Sequence | Length | S_AMP_ | Instability | pI | Charge | Hydrophobic moments | Aromaticity | Molecular weights |
| --- | --- | --- | --- | --- | --- | --- | --- | --- |
| MVGRA | 5 | 0.49 | -8.98 | 13.5508 | 1.99 | 0.7137 | 0 | 531.68 |
| RVRRVR | 6 | 0.6 | 103.8 | 13.8633 | 4.99 | 0.5682 | 0 | 840.04 |
| RVWRVRI | 7 | 0.56 | 8.5714 | 13.7969 | 3.99 | 0.3972 | 0.1429 | 983.22 |
| RVRIVRIR | 8 | 0.59 | -1.8625 | 13.8633 | 4.99 | 0.3862 | 0 | 1066.35 |
| RVRIVRIRR | 9 | 0.62 | 63.1 | 13.9141 | 5.99 | 0.4809 | 0 | 1222.54 |
| RVRIVRIRRV | 10 | 0.69 | 57.79 | 13.9141 | 5.99 | 0.5413 | 0 | 1321.67 |
| RVRIVRIRRVR | 11 | 0.71 | 53.4455 | 13.9550 | 6.99 | 0.5070 | 0 | 1477.85 |
| RVWIVRIRRVRG | 12 | 0.78 | 42.75 | 13.9141 | 5.99 | 0.4876 | 0.0833 | 1564.93 |
| RVRIVKIVRVRGR | 13 | 0.77 | 19.1077 | 13.9141 | 6.989 | 0.5609 | 0 | 1606.03 |
| RVGIVKIVRVLGRG | 14 | 0.96 | 29.1571 | 13.8008 | 4.989 | 0.7505 | 0 | 1520.92 |
| GVGIVKIGRILGRGR | 15 | 0.95 | 8.0467 | 13.8008 | 4.989 | 0.7121 | 0 | 1549.92 |
| WVGIVKIVRVLGRGRR | 16 | 0.96 | 6.23125 | 13.8633 | 5.989 | 0.7454 | 0.0625 | 1863.31 |
| RVGIVKIRRVLGRGRRR | 17 | 0.91 | 83.8294 | 13.9922 | 8.989 | 0.6414 | 0 | 2046.53 |
| CVCRVRVRRVRGVGRRVR | 18 | 0.88 | 59.4667 | 12.5762 | 8.682 | 0.5654 | 0 | 2181.69 |
| CVGRVRVRRVRGVGRRVRV | 19 | 0.91 | 52.3947 | 12.9409 | 8.836 | 0.5740 | 0 | 2234.73 |
| CVGRVRVRRVRGVGRRVRVC | 20 | 0.93 | 50.275 | 12.5762 | 8.682 | 0.5719 | 0 | 2337.87 |
| GVGIAKIGRILGVGRRGRSIR | 21 | 0.97 | 46.6810 | 13.9141 | 6.989 | 0.6464 | 0 | 2190.65 |
| GVLIHKIGKILGVGHPVRSIIG | 22 | 0.99 | 46.6545 | 13.5664 | 4.072 | 0.5440 | 0 | 2262.8 |
| GVLSAAIGAIAGVGHHAHSLIKR | 23 | 0.97 | 8.9739 | 13.5586 | 3.115 | 0.3068 | 0 | 2234.62 |
| GVLSALIGAIAGAGHHAHSLIKRK | 24 | 0.96 | 9.0167 | 13.5664 | 4.114 | 0.3144 | 0 | 2376.82 |
| GVLSALIGAIAGAGKHAHSAAKYKH | 25 | 0.97 | -3.984 | 11.0527 | 4.112 | 0.3191 | 0.04 | 2427.82 |
| GVLSKLIGKIAGAGKKAASSAKKKIS | 26 | 1 | 2.5692 | 13.2656 | 7.986 | 0.4888 | 0 | 2511.08 |
| GVLSKLIGKIAGAGAKAASSAVYKISG | 27 | 1 | 4.8963 | 11.1997 | 4.985 | 0.4528 | 0.0370 | 2516.99 |
| GVLSKKIGKIAGAGAKAASSAAKKISGH | 28 | 1 | 1.6714 | 13.2344 | 7.028 | 0.4813 | 0 | 2606.09 |
| GILSTAIGAIAGAGKSVLSSIKCKISGCC | 29 | 0.98 | 5.3724 | 9.7871 | 3.526 | 0.3243 | 0 | 2708.29 |
| GVLSTKIGSIAGAGASALSSIKSKISKSCC | 30 | 0.98 | 12.8033 | 10.6924 | 4.68 | 0.3340 | 0 | 2824.34 |
| GVLSTKIGSIAGAGASAASSILSKISKSCLC | 31 | 0.98 | 27.8774 | 10.4277 | 3.68 | 0.3645 | 0 | 2880.4 |
| SVCSCKISSILGCICPCTSSSVCSISGICVKC | 32 | 0.9 | 49.2375 | 7.9097 | 1.757 | 0.2501 | 0 | 3170.89 |
| SVCSCKICSILGPCCPCTSSSVCSISGICVKYC | 33 | 0.87 | 39.9273 | 7.8312 | 1.601 | 0.2150 | 0.0303 | 3334.09 |
| SVCSAKICSILGPCCPETSSSKYCISGCGVKYCS | 34 | 0.96 | 31.9147 | 7.9993 | 1.907 | 0.2353 | 0.0588 | 3464.1 |
| SVCIAKIPSILGPCCPETSSSKYCISGCGVKICRR | 35 | 0.97 | 49.9086 | 8.7185 | 4.063 | 0.2965 | 0.0286 | 3659.43 |
| SVCIAKIPSILGPCHPCCSSIKYCISGKGVKICCRK | 36 | 0.94 | 16.4472 | 9.1519 | 5.949 | 0.3414 | 0.02778 | 3795.74 |
| KVCIAKIGSILGPCHPCCSSIKYCISGCGLKICCRKK | 37 | 0.96 | 12.6243 | 9.1746 | 6.795 | 0.3231 | 0.0270 | 3913.94 |
| SVCIAKIGSILGPHHPCHSSIKYCISGYGLKIGSRKKN | 38 | 0.95 | 17.3737 | 10.4014 | 6.647 | 0.3246 | 0.0526 | 4052.82 |
| KVCIAKIGSICGPHHPCHSSIKYCISGYGLKCGSRKVCK | 39 | 0.96 | 10.0692 | 9.8057 | 7.184 | 0.3494 | 0.0513 | 4162.05 |
| SVCIAKIPSILGPHHPCHSSIKYCISGHGLKIGSRKVCKR | 40 | 0.93 | 28.78 | 10.586 | 7.536 | 0.3609 | 0.025 | 4311.2 |
| KVCIAKIPSILGPHHPCNSSIKYCISGKGLKIGSRKVCCRK | 41 | 0.94 | 8.7366 | 10.6073 | 9.298 | 0.3663 | 0.0244 | 4423.44 |
| KVCIAKIGSILGNGHPCNSSIKYCISGAGVKIGGRKGCCRKW | 42 | 0.98 | 0.9762 | 10.4775 | 8.256 | 0.3677 | 0.0476 | 4363.26 |
| KYCIAKIGSIWGNGCPCNSSWVYSISGAGVGIGGAWGNLWKWG | 43 | 0.83 | 2.9302 | 9.4824 | 3.522 | 0.2592 | 0.1628 | 4504.16 |
| KVCIAKIGSICGNGHPCNSSHVYCISGAGVKIGGAKGNLCKKWR | 44 | 0.98 | 6.6341 | 10.2241 | 7.298 | 0.24618 | 0.0455 | 4515.37 |
| KVCIAKIGSILGNGCPCNSSSVYKISGAGVKIGGAWGNLCKWGRW | 45 | 0.94 | 15.3356 | 10.3032 | 6.369 | 0.3427 | 0.0889 | 4639.48 |
| KKCIAKIGSILGCGGGCTCSIVCSISGAGVGIGGAIGGLIKKGRKC | 46 | 0.94 | 17.0217 | 10.0793 | 7.063 | 0.3089 | 0 | 4295.28 |
| KKCIAKIGSIAGVGGGATGSIVGSIAGAGVGIGGAIGGLICKGIKCC | 47 | 0.89 | 3.1936 | 10.1600 | 5.371 | 0.2948 | 0 | 4144.03 |
| KKKIAKIGSIAGVGAGGTASIVGSIAGAGVGIGGAIGGLIGKGIKCAC | 48 | 0.89 | 0.1271 | 10.9800 | 6.679 | 0.3024 | 0 | 4208.07 |
| KKKIAKIGSIAGGGAGGAASIVGSIAGAGVGIGGAIGGLIGKGIKKAKK | 49 | 0.91 | 1.8694 | 13.3125 | 9.985 | 0.3259 | 0 | 4314.2 |
| MKPIAKIGSIAGAGAGATGSIVGSIAGAGVGIGGAIGGLIGAGIKKADKK | 50 | 0.79 | -13.94 | 11.3740 | 5.987 | 0.28271 | 0 | 4388.21 |

**Table S12.** CPL-Diff results of generating AFP sequences of different lengths with the same initial noise. S_AFP_ denotes the AFP score obtained by the prediction tool on top of Antifungipept. S_AFP_ < 0.5 indicates that the prediction is non-AFP. S_AFP_ ≥ 0.5 indicates that the prediction is AFP.

| Sequence | Length | S_AFP_ | Instability | pI | Charge | Hydrophobic moments | Aromaticity | Molecular weights |
| --- | --- | --- | --- | --- | --- | --- | --- | --- |
| WRWRW | 5 | 0.99 | 237.12 | 13.707 | 2.99 | 0.0312 | 0.6 | 888.03 |
| WRWRWW | 6 | 0.99 | 199.2667 | 13.707 | 2.99 | 0.1497 | 0.6667 | 1074.24 |
| WRWRWWW | 7 | 1 | 172.2286 | 13.707 | 2.99 | 0.1707 | 0.7143 | 1260.45 |
| WRWRWWWR | 8 | 1 | 151.95 | 13.7969 | 3.99 | 0.3979 | 0.625 | 1416.64 |
| RVVRVKVRR | 9 | 1 | 59.9 | 13.8633 | 5.989 | 0.5661 | 0 | 1166.47 |
| RVRRVKIRRV | 10 | 0.99 | 112.19 | 13.9141 | 6.989 | 0.3746 | 0 | 1336.68 |
| RVGIVKIRRIL | 11 | 0.99 | 45.1818 | 13.8008 | 4.989 | 0.5101 | 0 | 1321.71 |
| KVGIAKIKRILG | 12 | 0.98 | 17.0083 | 13.5742 | 4.988 | 0.5915 | 0 | 1294.68 |
| GVLIAKAGKILGK | 13 | 0.99 | 4.4538 | 13.0781 | 3.988 | 0.5111 | 0 | 1266.63 |
| GLLSAIIGKIAGLG | 14 | 0.97 | -2.8429 | 12.8438 | 1.989 | 0.397 | 0 | 1281.6 |
| GLLSALIGKIAGVIG | 15 | 1 | -1.9867 | 12.8438 | 1.989 | 0.4065 | 0 | 1380.73 |
| GLLSALAGKIAGVGGL | 16 | 0.94 | 1.1687 | 12.8438 | 1.989 | 0.3039 | 0 | 1395.7 |
| GVLSAKIGKILGKGGPG | 17 | 0.81 | 3.0294 | 13.0781 | 3.988 | 0.4837 | 0 | 1550.9 |
| GALIAKIGRILGGGRRGR | 18 | 0.96 | 51.5278 | 13.8633 | 5.989 | 0.6412 | 0 | 1820.21 |
| GVLSALIGKIAGIGGPARS | 19 | 0.96 | 35.8263 | 13.5586 | 2.989 | 0.3622 | 0 | 1736.08 |
| GVLSKLIGAIAGVGGAAKSL | 20 | 0.95 | -1.31 | 13 | 2.989 | 0.3508 | 0 | 1781.16 |
| GVLSKLIGAIKGVGGAASSLI | 21 | 1 | 0.3143 | 13 | 2.989 | 0.4007 | 0 | 1910.32 |
| GLLSHLIGAIKGAGKHAHSLIK | 22 | 1 | -17.4682 | 13.0781 | 4.114 | 0.4502 | 0 | 2220.68 |
| GVLSHLIGAIAGAGKHAHSLIKR | 23 | 1 | 5.2826 | 13.5664 | 4.114 | 0.3986 | 0 | 2305.74 |
| GVLSHLIGAIAGAGKHAHSLAKRR | 24 | 1 | 32.8833 | 13.7148 | 5.114 | 0.3783 | 0 | 2419.85 |
| GVLSKLIGKIAGAGKHALSAAKYKL | 25 | 1 | -10.776 | 11.3105 | 6.027 | 0.4275 | 0.04 | 2494.05 |
| GALSSLIGKIAGAGKHALSAAKKKLS | 26 | 1 | -2.5692 | 13.1875 | 6.029 | 0.4113 | 0 | 2476.98 |
| GALSSLIGKIAGAGKKALSAAAKKLSG | 27 | 1 | -2.1037 | 13.1875 | 5.987 | 0.4563 | 0 | 2467.97 |
| GLLSSLIGKILGAGKKALSAAKGKLSGK | 28 | 1 | -0.8571 | 13.2344 | 6.986 | 0.3817 | 0 | 2666.27 |
| GLLSTLIGKILGAGKSALSSAVGKLSGAG | 29 | 1 | -0.4828 | 13.0781 | 3.988 | 0.3991 | 0 | 2626.12 |
| GVLSTAIGKIAGVAKNALGSIVCKISGGGC | 30 | 0.99 | 6.5733 | 10.4277 | 3.68 | 0.4205 | 0 | 2744.3 |
| GVLSTLKGKIAGAGKSALSSIKSKLSGSGCC | 31 | 0.99 | -6.0194 | 10.8591 | 5.679 | 0.2961 | 0 | 2908.46 |
| GVLSTLKGAIKGAGKSAASSLLSKLSCKLCKC | 32 | 1 | 2.0156 | 10.686 | 6.525 | 0.4634 | 0 | 3122.83 |
| GILSTLKGAILGAGKSAASSALSKLSCKLLKTC | 33 | 1 | 13.9303 | 10.8591 | 5.679 | 0.392 | 0 | 3190.88 |
| GILSTAIGSILGAGKNAASSAVKKLSKCLLKTTC | 34 | 1 | 19.0324 | 10.8591 | 5.679 | 0.4642 | 0 | 3304.98 |
| GILSTKIGSILGVGGKAASSAVYKLSKKLLKTTSR | 35 | 1 | 16.1171 | 11.8735 | 7.984 | 0.4528 | 0.0286 | 3533.23 |
| SSGSHKYGSYYGKGHHYHSSSKYRGSGYGYYYYYRY | 36 | 0.96 | 34.7667 | 9.918 | 6.131 | 0.225 | 0.3333 | 4316.56 |
| SSCSYKSGSYYGYCRPYRSSSGYRGSGYGYYYYYRYG | 37 | 0.98 | 33.8081 | 9.6354 | 5.655 | 0.3296 | 0.3514 | 4377.68 |
| SSCSYKSGSYYGYCRPYRSSSGYRGSGYGYYYYSRYGY | 38 | 0.95 | 37.2184 | 9.6354 | 5.655 | 0.3338 | 0.3421 | 4464.76 |
| SVCSAKSGSYYGPHHPCRSSSGRRGSGYGLYYGSRKYCY | 39 | 0.22 | 43.2846 | 9.9517 | 6.596 | 0.3166 | 0.1795 | 4284.71 |
| SVCSAKSCSIYGPHHPCRSSSRRRGSGYGLCYGSRKVCCR | 40 | 0.17 | 61.445 | 10.1777 | 8.142 | 0.3116 | 0.075 | 4352 |
| GSCSAKSSSICGNHHPCRSSSKRRGSGYGVCYGKRKVCCRN | 41 | 0.98 | 63.061 | 10.4807 | 9.143 | 0.2861 | 0.0488 | 4363.97 |
| AVCSAKSGSVCGNHAPCNSSSKRRGSGYGLGYGGRKVCCRRW | 42 | 0.89 | 59.1881 | 10.6072 | 8.256 | 0.297 | 0.0714 | 4362.96 |
| AVCSAKSGSVCGNAAHCNCSSKRCISGYGLGYGGRKVCCRNWK | 43 | 0.96 | 10.6651 | 9.5247 | 6.948 | 0.3286 | 0.0698 | 4442.14 |
| AACSAKSSSVFGNKCPCNASAKRCISGYGLGYGGRKVCVCNCRR | 44 | 0.96 | 45.8455 | 9.8906 | 7.906 | 0.3305 | 0.0682 | 4533.29 |
| KVCDAKSSSVCGNCCDANCSAKHCISGYGLGYGGRKVNKRNCCRT | 45 | 1 | 29.7622 | 8.8193 | 5.794 | 0.3033 | 0.0444 | 4717.42 |
| KVCDAKSSSVCGNCTAANCSAKHCASGHGLGYGGRKGNRRNWCRCN | 46 | 0.91 | 30.413 | 9.6206 | 6.992 | 0.3135 | 0.0435 | 4788.4 |
| KVCDAKSSSVFGNGTTCNSSAKHCASKHGKGYGGRKGNLRNWCRCQN | 47 | 0.97 | 10.0553 | 10.4719 | 8.298 | 0.3396 | 0.0638 | 5003.61 |
| KVCKAKSSSVFGNGTTCNSSSKHCISHHGLGYGGRKGCLRNWCTCCAK | 48 | 0.96 | 27.7354 | 9.9272 | 8.032 | 0.3145 | 0.0625 | 5062.85 |
| KVCKAKSSSVLGNGCTCNSSSKHCISKHGLGYGGRKGCLRNWCTCCTDT | 49 | 0.95 | 39.3449 | 9.1743 | 6.837 | 0.3268 | 0.0408 | 5139.95 |
| KSCCAKSCSVCGCGCTCNSSSKKCKSHHGLKCGGRKGCLRNWGRCCTDTT | 50 | 0.87 | 43.6 | 8.7694 | 8.222 | 0.3716 | 0.02 | 5234.15 |

**Table S13.** CPL-Diff results of generating AVP sequences of different lengths with the same initial noise. SAVP denotes the AVP score obtained by the prediction tool on top of Srack-AVP. S_AVP_ < 0.5 indicates that the prediction is non-AVP. S_AVP_ ≥ 0.5 indicates that the prediction is AVP.

| Sequence | Length | S_AVP_ | Instability | pI | Charge | Hydrophobic moments | Aromaticity | Molecular weights |
| --- | --- | --- | --- | --- | --- | --- | --- | --- |
| SCLSC | 5 | 0.99 | 176.92 | 8.0967 | 0.682 | 0.2319 | 0 | 510.63 |
| SCSSSC | 6 | 0.88 | 181.2 | 8.0967 | 0.682 | 0.1281 | 0 | 571.63 |
| CCASTAW | 7 | 0.97 | 8.5714 | 8.0967 | 0.682 | 0.1936 | 0.1429 | 739.87 |
| CCASTAWC | 8 | 0.955 | 8.75 | 7.8213 | 0.528 | 0.1957 | 0.125 | 843.01 |
| CCASTSWLC | 9 | 1 | 22.6 | 7.8213 | 0.528 | 0.141 | 0.1111 | 972.17 |
| SCASTAWLSR | 10 | 0.425 | 73.2 | 10.7539 | 1.836 | 0.2254 | 0.1 | 1080.23 |
| TAASTAWLSRA | 11 | 0.62 | 37.8182 | 13.5508 | 1.99 | 0.264 | 0.0909 | 1133.26 |
| KKKKVVAATVVV | 12 | 1 | 2.0917 | 13.1406 | 4.987 | 0.2347 | 0 | 1268.64 |
| KKKKVVAATVVVV | 13 | 1 | 2.7 | 13.1406 | 4.987 | 0.213 | 0 | 1367.77 |
| KKKKVVVATVVVVV | 14 | 0.995 | 3.2214 | 13.1406 | 4.987 | 0.2087 | 0 | 1494.96 |
| VPASTFWLSVAVSAA | 15 | 0.57 | 51.4667 | 12.25 | 0.99 | 0.1903 | 0.1333 | 1504.74 |
| VPASTFWLSVWVSAAV | 16 | 0.675 | 43.5688 | 12.25 | 0.99 | 0.1949 | 0.1875 | 1719 |
| VPASTIWLSVWWSAAVS | 17 | 0.795 | 39.3294 | 12.25 | 0.99 | 0.2093 | 0.1765 | 1859.14 |
| TPASTIWLSVWWSVAVSA | 18 | 0.715 | 27 | 12.25 | 0.99 | 0.2278 | 0.1667 | 1960.24 |
| TPASTIWLSVWVSAAVSEC | 19 | 0.685 | 54.9 | 6.125 | -0.164 | 0.2299 | 0.1053 | 2006.29 |
| TPASTIWLSVSVSVAVSEWV | 20 | 0.56 | 23.17 | 6.75 | -0.01 | 0.1886 | 0.1 | 2117.41 |
| TPASTIWLSVSVSVAVSELVN | 21 | 0.08 | 33.7429 | 6.75 | -0.01 | 0.2178 | 0.0476 | 2158.46 |
| FPASTIWLSVSVAVAVSELVNI | 22 | 0.095 | 61.3909 | 6.75 | -0.01 | 0.2176 | 0.0909 | 2301.69 |
| FLLSFIWVSVSVAPAVSALVSIL | 23 | 0.445 | 30.9957 | 12.25 | 0.99 | 0.2136 | 0.1304 | 2417.94 |
| FLLSVIGVAVSVAPAVICAVNSHF | 24 | 0.625 | 21.3125 | 8.7627 | 0.878 | 0.2388 | 0.0833 | 2412.9 |
| FLLSVIWVAVSVAPAVIPAVISHFA | 25 | 0.455 | 24.016 | 12.25 | 1.031 | 0.2509 | 0.12 | 2606.16 |
| FLPSFIWLLVSVAKAVIPAVIIHFAK | 26 | 0.135 | 36.2346 | 13 | 3.03 | 0.3325 | 0.1538 | 2879.58 |
| FLASTIALLVSVAKAVIAAVNIHFAKI | 27 | 0.69 | 23.4889 | 13 | 3.03 | 0.2712 | 0.0741 | 2780.41 |
| FIASTIALLGSVAKAVIPPVIIGFAKIL | 28 | 0.1 | 26.2179 | 13 | 2.989 | 0.3329 | 0.0714 | 2822.53 |
| TPASTIWLSVAVAAATIAAVNIGFAKILG | 29 | 0.15 | 39.4172 | 12.8438 | 1.989 | 0.1843 | 0.069 | 2826.35 |
| TPASTIWLSRAVAKAVIEAVNIGFAKILGV | 30 | 0.095 | 59.5033 | 11.5723 | 2.989 | 0.423 | 0.0667 | 3095.69 |
| SPASTIALSVSVAKATIEAVNIGFAKILGVS | 31 | 0.105 | 61.8871 | 10.709 | 1.989 | 0.2793 | 0.0323 | 3014.53 |
| SDLSTIEKSVSSAKATIESVNQSIAKILDVLS | 32 | 0.295 | 63.9281 | 6.9375 | -0.01 | 0.4653 | 0 | 3333.76 |
| SELSSIEKSIKSAEATIESVNSSIAKILAVLSS | 33 | 0.385 | 66.2 | 7.0313 | -0.01 | 0.4504 | 0 | 3391.83 |
| SELSSIEKSIKSAEATIESVNSSIAKILAVLSSS | 34 | 0.34 | 70.2118 | 7.0313 | -0.01 | 0.4394 | 0 | 3478.91 |
| SELSSIEKSIKSANATIESVNSSIAKILAVLSSSD | 35 | 0.34 | 68.4914 | 7 | -0.01 | 0.4316 | 0 | 3578.99 |
| SELSSIEKSIKSAVATIESVNQSIAKILAVLSSSDQ | 36 | 0.36 | 71.6278 | 7 | -0.01 | 0.4592 | 0 | 3733.2 |
| SELSTIEKSIKDAEATIESVNSSIAKILAVLSSSDQS | 37 | 0.835 | 72 | 4.3228 | -2.009 | 0.4291 | 0 | 3851.24 |
| SELSSIEKSIKDAEATIESVNSSIAKILAVLSSSDGSI | 38 | 0.27 | 63.8737 | 4.3228 | -2.009 | 0.4095 | 0 | 3879.3 |
| SDLSTINKSIKDAVATIESVNSSIAKILAVLSSSDGSSV | 39 | 0.15 | 52.359 | 6.875 | -0.011 | 0.4195 | 0 | 3907.36 |
| SELSTINKSIKDAVATIESVNSSIAKILAVLSSSDGSLVR | 40 | 0.23 | 51.3 | 9.7261 | 0.99 | 0.4144 | 0 | 4103.65 |
| SELSTINKSIRDAVATIESVNNSIAKILAVLDSSDGSLVRN | 41 | 0.325 | 50.6756 | 7 | -0.01 | 0.4636 | 0 | 4300.8 |
| SDLSTINKSIRDAVATIESVNSSIAKILAVLDSSDGSLVRNV | 42 | 0.17 | 49.7071 | 6.9375 | -0.01 | 0.4535 | 0 | 4358.88 |
| SDLSTINKSINDAVATIESVNSSIAKILAVLNSSDGSLVRNVN | 43 | 0.15 | 44.3047 | 6.875 | -0.01 | 0.4336 | 0 | 4429.91 |
| SDLSTINKSINDAVATIESVNDSIAKILAVLNSSDGSLVRNVNS | 44 | 0.185 | 43.525 | 4.4209 | -1.01 | 0.4605 | 0 | 4545 |
| MDLSTINKSIRDAVATIESVNDSIAKILAVLNSSDGSLVRNIRSL | 45 | 0.12 | 62.3089 | 9.8232 | 0.99 | 0.5006 | 0 | 4800.47 |
| ADLSTINVSIRNAVATIESVNASIAKILAVLNSSVGSLVRNVNSLL | 46 | 0.335 | 31.7239 | 11.2207 | 1.99 | 0.4223 | 0 | 4707.4 |
| SDLSTINKSIRNAVATIESVNASIAKILAVLGSSVGSLVRNVNSLLK | 47 | 0.3 | 36.3 | 11.4951 | 3.989 | 0.4864 | 0 | 4823.57 |
| SDLSTIAKSINNAVATIESVNASIAKILAVLGSSLGSLVRNVNSLLGA | 48 | 0.155 | 30.0167 | 10.6797 | 1.989 | 0.4628 | 0 | 4752.45 |
| SDLSTINKSINNAVATIESVNASIAKILAVLGSSLGSLVRNVNSLLGAL | 49 | 0.18 | 34.4408 | 10.6797 | 1.989 | 0.4524 | 0 | 4908.63 |
| SDLSTINKEIANAVATIENVNQSIAKILAVLGSSLGSLVNNVQSLLGALA | 50 | 0.35 | 47.552 | 6.9375 | -0.01 | 0.4476 | 0 | 5034.74 |
